# Ruthenium Tetroxide and Perruthenate Chemistry. Recent Advances and Related Transformations Mediated by Other Transition Metal Oxo-species

**DOI:** 10.3390/molecules19056534

**Published:** 2014-05-21

**Authors:** Vincenzo Piccialli

**Affiliations:** Dipartimento di Scienze Chimiche, Università degli Studi di Napoli “Federico II”, Via Cintia 4, 80126, Napoli, Italy; E-Mail: vincenzo.piccialli@unina.it; Tel.: +39-081-674111; Fax: +39-081-674393

**Keywords:** ruthenium tetroxide, perruthenate, TPAP, oxidation

## Abstract

In the last years ruthenium tetroxide is increasingly being used in organic synthesis. Thanks to the fine tuning of the reaction conditions, including pH control of the medium and the use of a wider range of co-oxidants, this species has proven to be a reagent able to catalyse useful synthetic transformations which are either a valuable alternative to established methods or even, in some cases, the method of choice. Protocols for oxidation of hydrocarbons, oxidative cleavage of C–C double bonds, even stopping the process at the aldehyde stage, oxidative cleavage of terminal and internal alkynes, oxidation of alcohols to carboxylic acids, dihydroxylation of alkenes, oxidative degradation of phenyl and other heteroaromatic nuclei, oxidative cyclization of dienes, have now reached a good level of improvement and are more and more included into complex synthetic sequences. The perruthenate ion is a ruthenium (VII) oxo-species. Since its introduction in the mid-eighties, tetrapropylammonium perruthenate (TPAP) has reached a great popularity among organic chemists and it is mostly employed in catalytic amounts in conjunction with *N*-methylmorpholine *N*-oxide (NMO) for the mild oxidation of primary and secondary alcohols to carbonyl compounds. Its use in the oxidation of other functionalities is known and recently, its utility in new synthetic transformations has been demonstrated. New processes, synthetic applications, theoretical studies and unusual transformations, published in the last eight years (2006–2013), in the chemistry of these two oxo-species, will be covered in this review with the aim of offering a clear picture of their reactivity. When appropriate, related oxidative transformations mediated by other metal oxo-species will be presented to highlight similarities and differences. An historical overview of some aspects of the ruthenium tetroxide chemistry will be presented as well.

## 1. Introduction

Oxidation is one of the fundamental reactions in synthetic organic chemistry both in academia and industry and there is always demand for selective and mild oxidation methods. Indeed, numerous research groups have directed their efforts towards the development of novel oxidation processes and the trend is relentless. In particular, significant progress has been achieved within the area of catalytic oxidations. In this regard transition metal oxo-species such as OsO_4_ [1,2], RuO_4_, RuO_4_^−^, MnO_4_^−^ [3,4,5], rhenium [6,7] and references therein] and chromium oxo-species [8], play a primary role since they catalyze a wide spectrum of synthetically useful oxidative transformations [9,10].

## 2. Ruthenium Tetroxide Chemistry

### 2.1. An Historical Overview

It is recognised that ruthenium tetroxide [11,12,13,14,15] was introduced as an organic oxidant in 1953 by Djerassi and Engle [16] who used this reagent to oxidise phenanthrene and a range of sulfides. The oxidation of a variety of compounds ensued [17] generally using catalytic amounts of a ruthenium precatalyst, usually RuCl_3_ hydrate or ruthenium dioxide, in the presence of a co-oxidant [18]. These processes were often reported to be slow or incomplete generally due to the precipitation of ruthenium-containing species. It was not until the report by Sharpless and co-workers [19], almost thirty years later, that a new effective catalytic oxidative protocol was introduced. In particular, based upon the assumption that carboxylic acids, generated during the oxidation process or already present in solution, could inactivate the ruthenium catalyst, these authors added CH_3_CN to the solvent mixture to disrupt the insoluble low-valent ruthenium-carboxylate complexes and reactivate the catalytic cycle. With the new solvent system, CCl_4_/CH_3_CN/H_2_O (2:2:3), the effective catalytic oxidation of a variety of organic compounds was carried out in the presence of sodium metaperiodate (4.1 equiv.) and ruthenium trichloride hydrate (2.2 mol%). Since then these experimental conditions are known as “Sharpless conditions” and have been used for the oxidation of a range of functional groups [20,21,22,23,24,25,26,27,28,29,30,31,32]. For example, these are often the preferred conditions for cleavage of electron-poor C-C double bonds when the two-step process using osmium tetroxide (diol formation and successive diol cleavage) fails. However, the subsequent use of RuO_4_ in organic synthesis was hampered by the perception that it was too a strong oxidant to be also selective and useful for oxidative transformations when used with multifunctional molecules. As new evidence about the reactivity of RuO_4_ grew up, this idea proved wrong in several instances. Although a number of researchers have contributed to the advance of the knowledge about the RuO_4_ chemistry, it is thanks to the systematic studies of a few research groups that effective oxidative protocols have been developed and are now increasingly employed in synthesis. 

One of the most known, and employed, reaction catalyzed by RuO_4_ is the oxidative cleavage of carbon-carbon double bonds. Since the seminal contribution by Sharpless and co-workers [19], new protocols have been developed [33,34,35,36] and the oxidative cleavage of olefins, even stopping the process at the aldehyde stage, is now feasible [37]. A related oxidative cleavage of terminal and internal alkynes [38,39,40] was developed as well, to give carboxylic acids or ketones [41].

**Scheme 1:**
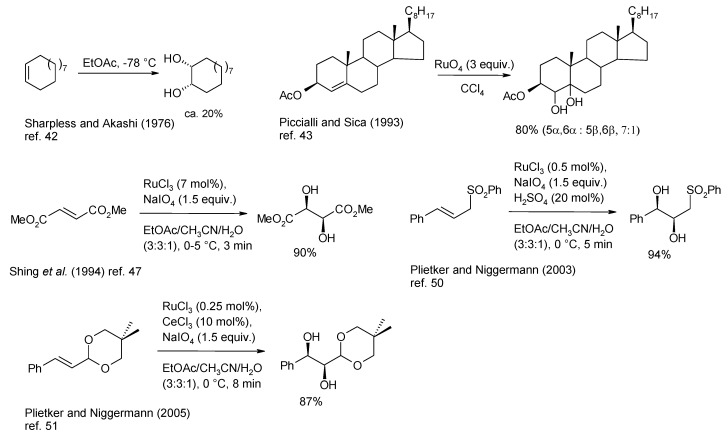
The evolution of the dihydroxylation of alkenes through representative examples.

The ruthenium tetroxide dihydroxylation of alkenes has been the subject of various improvements in the last twenty years. Rather surprisingly, since the first report on the *cis*-dihydroxylation of (*E*)- and (*Z*)-cyclododecene by Sharpless and Akashy [42] (Scheme 1), this process has long been neglected probably due to the poor yields and the comment of these authors about the process that they considered “not a practical route to diols”, as well as to the great attention devoted to the analogous osmium tetroxide-catalyzed process.

In 1993 the group of Piccialli and Sica employed stoichiometric amounts of RuO_4_ to oxidise a range of steroidal alkenes [43,44] and dienes [45] demonstrating for the first time that good yields of *syn*-diols could be obtained (Scheme 1), and therefore that the behaviour of this oxide resembled that of OsO_4_ more than previously believed. Notably, referring to these studies, the change of solvent used in the process from acetone-water to CCl_4_ resulted in the switch of the reaction products from α-ketols, obtained in the former solvent mixture, to 1,2-diols. Indeed, in most cases, the oxidative behaviour of RuO_4_ is strongly affected by the employed solvent. In the course of these studies Piccialli and co-workers were able to isolate for the first time the ruthenium (VI) diester intermediate [46] (see later for a more detailed discussion), formed by reaction of RuO_4_ with two molecules of 7-dehydrocholesteryl acetate.

Subsequently Shing and co-workers [47,48,49] rendered the dihydroxylation process catalytic and coined the term “flash dihydroxylation” for this transformation due to the very short times required (Scheme 1). Since this contribution, the Shing’s protocol has been employed in several instances. More recently Plietker and co-workers were able to further improve this transformation conducting the process under acidic conditions, by addition of catalytic amounts of a Bronstedt acid [50] or by the addition of CeCl_3_ [51] to the reaction mixture (Scheme 1). The use of this Lewis acid allowed a decrease of the catalyst loading up to 0.25 mol% and, importantly, the short times rendered various acid-labile functional groups, including acetals, tolerant to the reaction conditions, and further increased the yields of the process. At the moment this represents the most reliable dihydroxylating system based on ruthenium tetroxide and is increasingly used in synthesis.

In the meantime, some other important processes were developed namely the stereoselective oxidative cyclization of 1,5-dienes [52,53], 1,6-dienes [54] and 1,7-dienes [55,56] to *cis*-THF, *trans*-THP and *trans*-oxepanes, respectively, addressed by Piccialli and co-workers and the stereoselective oxidative polycyclization of polyenes [57,58,59,60] to poly-THF compounds, studied by the same group. While formation of THF diols from 1,5-dienes had been recorded by Sharpless and co-workers some years before [19], the synthesis of THP and oxepanes constituted an advance of this type of process. THF [61,62] and THP-forming [63] processes were later revisited by Stark and co-workers and the development of new protocols was carried out.

**Figure 1:**
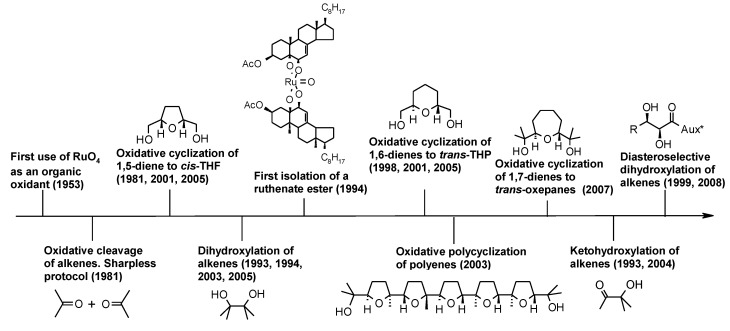
Chronological development of the oxidative transformations of alkenes, dienes and polyenes catalysed by RuO_4_. Significant dates are shown in parentheses.

Next, the ketohydroxylation of alkenes [64,65,66,67] and the regioselective monooxidation of *vic*-diols to enantiopure α-ketols [67,68] were addressed by Plietker and co-workers. Although the preparation of α-ketols from alkenes had previously been accomplished under classical conditions, mostly for trisubstituted alkenes [43,45,69], a turning point in the ketohydroxylation of alkenes was represented by the introduction of oxone, as a nucleophilic reoxidant, in the process [64]. In 2004 Plietker published a paper where the RuO_4_-catalysed dihydroxylation and ketohydroxylation processes, as well as the mono oxidation of 1,2-diols, were fully treated [70] and, in 2005 a comprehensive review on the RuO_4_ chemistry [71], whereas in 2007 Piccialli reviewed the oxidative cyclization of dienes and the polycyclization of polyenes catalysed by RuO_4_ [72]. The asymmetric dihydroxylation of alkenes has been the subject of a few but important contributions [73,74]. Indeed, the most recent advance in the RuO_4_ chemistry, due once again to Plietker’s group, is represented by the diasteroselective dihydroxylation of alkenes carrying suitable chiral auxiliaries. The chronological development of the oxidative transformations of alkenes, dienes and polyenes, mediated by RuO_4_ are summarized in Figure 1.

Accounts of some aspects of the RuO_4_ chemistry have previously been published [75,76,77,78] but no comprehensive review has been published in the last years. In the present account we report an up-to-date picture of the RuO_4_ chemistry covering the literature in the period 2006-2013, with a special focus on the oxidative chemistry of alkenes and polyenes, by also discussing some aspects of the relevant processes not fully dealt with previously. Mechanistic aspects of the RuO_4_-mediated transformations have been reported in the previous Plietker’s review [71] and they will not be further discussed here if not specifically required. New promising transformations, such as the oxidative spiroketalization of some ω-hydroxy-THFs [79] and a few other interesting, though isolated, results will be reported as well, to give an as clearer as possible picture of the ruthenium tetroxide reactivity. Our aim is to stimulate further interest in the RuO_4_ chemistry so that further studies, and hopefully new progresses, could result.

### 2.2. Diastereoselective Dihydroxylation of Alkenes and Tandem RCM/ Oxidation or CM/Oxidation Processes

**Scheme 2:**
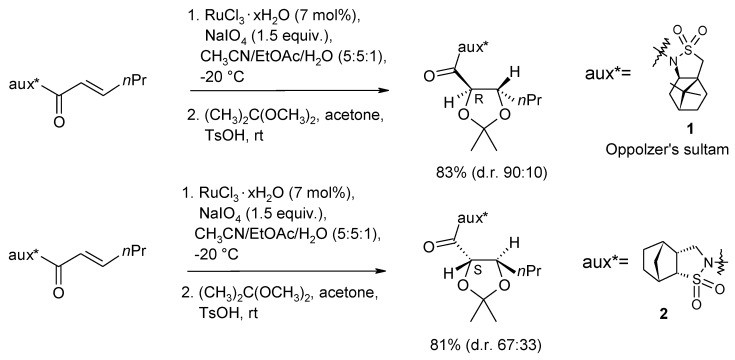
Examples of diastereoselective dihydroxylation of α,β-unsaturated carbonyl compounds (major diastereomers shown) [73].

The first examples of diastereoselective dihydroxylation of olefins catalyzed by RuO_4_ were reported in 1999 by Lee and co-workers [73]. Oxidation of enoyl derivatives of the Oppolzer’s camphorsultam auxiliary 1 (Scheme 2), using the Shing’s dihydroxylation protocol [48], gave good yields of diols and an up to 9:1 selectivity was observed. The sense of diastereoselectivity was the same observed in the analogous OsO_4_-catalyzed process. An opposite sense of diastereoselectivity was observed with the related sultam 2, but a poorer *ca* 6:4 selectivity was generally observed.

**Scheme 3:**
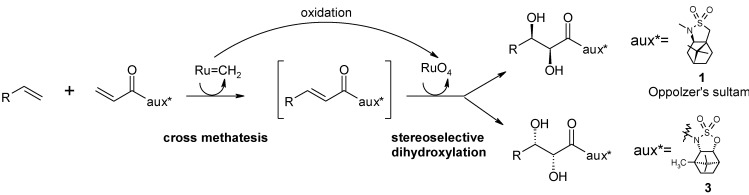
Sequential CM/dihydroxylation developed by Neisius and Plietker [74].

More recently, Neisius and Plietker have carried out a thorough investigation of the diastereoselective dihydroxylation of olefins developing an auxiliary-based diasteroselective sequential cross metathesis(CM)/dihydroxylation protocol [74] (Scheme 3). It is based on the concept of sequential catalysis: the use of a single catalytic source to mediate two or more different sequential reactions. Initially, the dihydroxylation of α,β-unsaturated corboxamides, using various enantiopure oxazolidinones as chiral auxiliaries, was addressed. Importantly, a reversal π-facial selectivity was observed compared to the Oppolzer’s camphorsultam-based dihydroxylation previously reported Lee and co-workers [73], allowing the access to diols of the opposite enantiomeric series.

Yields were good, but the desired products were obtained with moderate diastereoselectivities. Better results were obtained with a new, *ad hoc* synthesized, camphor-derived sulfamidate auxiliary 3 (Scheme 3) that gave diols in good yields and the desired opposite sense of stereoinduction. In this sense the new auxiliary could be considered a “pseudoenantiomeric camphorsultam”. A comparison with the results obtained by the same group on a range of substrates, using the Oppolzer’s sultam auxiliary, proved that the new developed auxiliary gave higher diatereolectivities (d.r. up to 12:1; best d.r. with the Oppolzer’s sultam auxiliary 9:1).

**Scheme 4:**
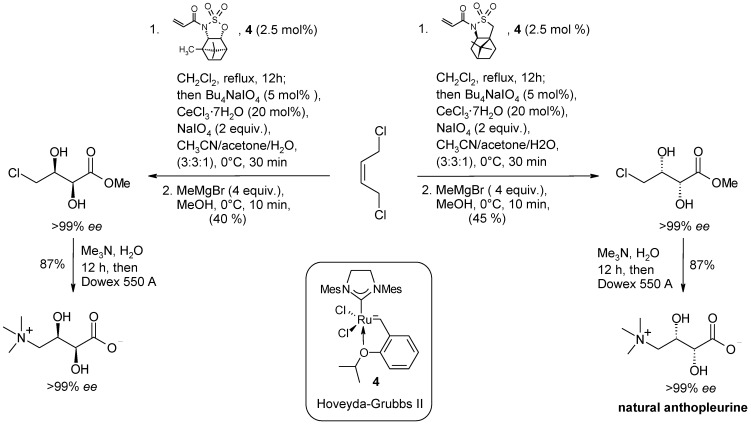
Synthesis of both the enantiomers of anthopleurine by sequential Ru-catalyzed CM/dihydroxylation [74].

Next, the sequential CM/dihydroxylation protocol was addressed. The use of various catalysts showed that the highest catalytic activity in the CM process was displayed by the Hoveida-Grubbs II complex 4 (Scheme 4) that, importantly, was also active as a ruthenium source in the subsequent dihydroxylation step. An investigation of the influence of some additives in this step showed that the addition of Bu_4_NIO_4_ in acetone allowed the obtaining of diols in good yields though with moderate diastereselectivity. The developed whole catalytic sequence was shown to be broadly applicable and the selectivities obtained in the dihydroxylation step significantly improved in the final methanolysis step. Overall, the diols were obtained with enantiomeric excesses in the range 91%–99%. The synthesis of both the enantiomers of anthopleurine, an alarm pheromone isolated from the sea anemone *Anthopleura elegantissima* (Scheme 4), was carried out using the new protocol. This synthesis also highlights the whole developed CM/dihydroxylation sequence.

**Scheme 5:**
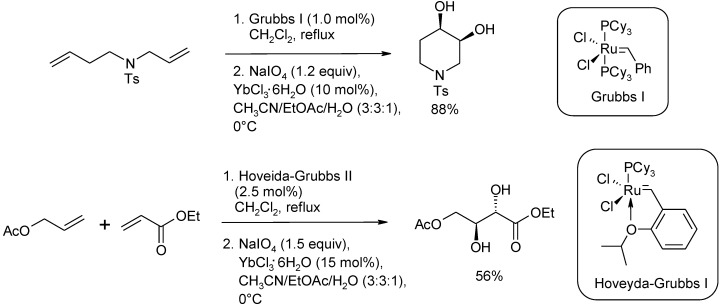
RCM/dihydroxylation and CM/dihydroxylation protocols developed by Blechert and co-workers [80].

Independently, Blechert and co-workers [80] developed related Ru-mediated sequential RCM/dihydroxylation and CM/dihydroxylation protocols using Grubbs I and Hoveyda-Grubbs I precatalysts, respectively. The addition of a 10–15 mol% amount of YbCl_3_•6H_2_O was required for the process. Selected examples are shown in Scheme 5. This represents the first use of a Ru-carbene species as a source of RuO_4_ in the dihydroxylation of alkenes.

**Scheme 6:**
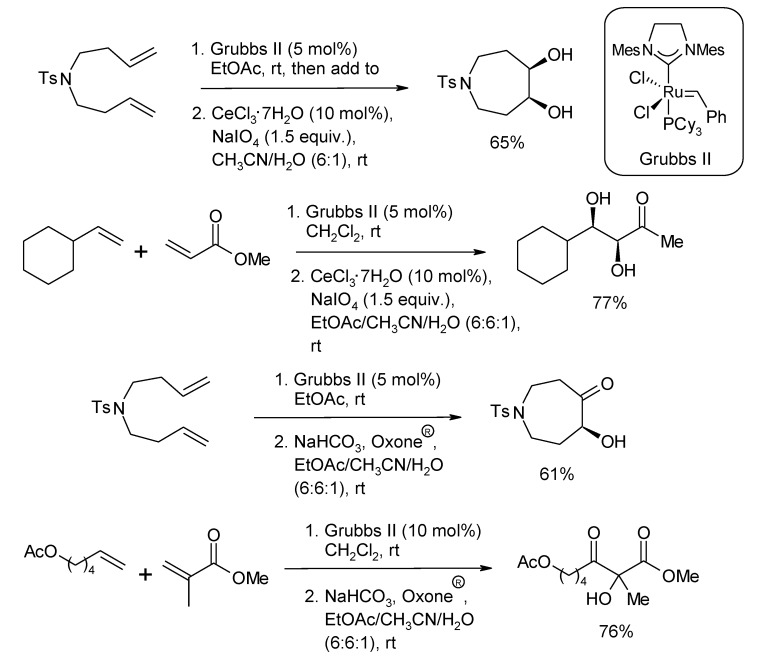
RCM or CM/dihydroxylation and RCM or CM/α-ketohydroxylation protocols developed by Snapper and co-workers [81].

Similarly, shortly after the Blechert’s report, Snapper and co-workers [81] developed two ruthenium-catalyzed protocols consisting of a RCM or CM step followed by either a Ru-catalyzed dihydroxylation or a Ru-catalyzed α-ketohydroxylation, generally using a 5 mol% of Grubbs II pre-catalyst, under Plietker’s conditions. Selected examples of such processes are shown in Scheme 6. Yields for the RCM/dihydroxylation process were in the range 63%–81% and five-, six-, and seven membered diols could be obtained. The reaction displayed high diastereoselectivity when a nearby stereocenter was present. The CM/dihydroxylation process proceeded with 42%–77% yields and could be performed in a variety of solvents. Even hindered diols, obtained from trisubstituted olefins, could be accessed in good yields with this process. The tandem RCM/α-ketohydroxylation process gave cyclic α-ketols in 42%–61% yields. In some cases a 10 mol% amount of Grubbs II catalyst was required. The CM/α-ketohydroxylation sequence proceeded in 49%–76% yields but the regioselectivity of the oxidation step was generally low.

**Scheme 7:**
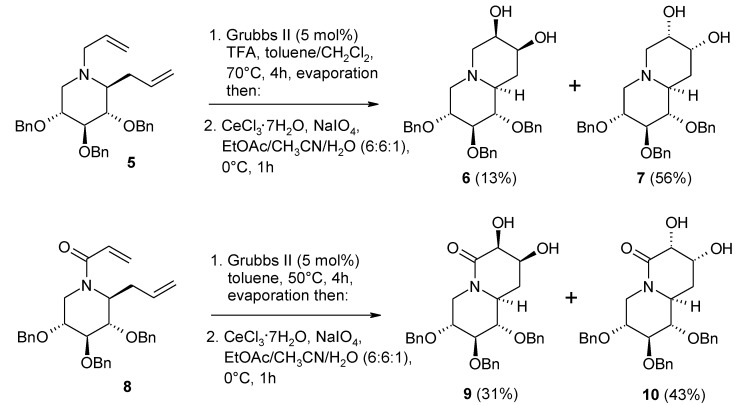
Tandem RCM/dihydroxylation in the synthesis of polyhydroxylated quinolizidines [82].

The synthetic potential of the above RCM/dihydroxylation sequence for the synthesis of functionalized five to seven-membered rings is evident. Recently, for example, it has been used in the synthesis of some polyhydroxylated quinolizidines [82] (Scheme 7). Starting from the bis-allyl compound 5, the expected bicyclic diol was obtained in 69% yield as a mixture of the diastereomers **6** and **7** (**6/7**, 1:4.3). Since amines are known to deactivate the catalyst in olefin methatesis, the RCM step was accomplished in the presence of TFA that converts the amine into the TFA salt *in situ*. Interestingly, the analogous OsO_4_-catalyzed dihydroxylation of the first-formed olefin gave a reversed ratio of the two diastereomers (**6/7**, 3.5:1). Similarly, the related acryloyl substrate 8 gave the desired diol **9/10** in a good overall yield (74%) but poor diastereoselectivity (**9/10**, 1:1.4). In this case no TFA was require for the RCM step.

### 2.3. Synthetic Applications of the Ru-Catalyzed Dihydroxylation of Olefins

Although novel protocols for the dihydroxylation of olefins have been developed over recent years [77], the OsO_4_-catalyzed process is still the most used for this transformation and its asymmetric version is one of the most important processes ever developed in synthesis [83]. However, sometimes this route is not effective whereas the related process catalyzed by RuO_4_ is successfully employed. Some synthetic application of the latter process are presented in this section.

**Scheme 8:**
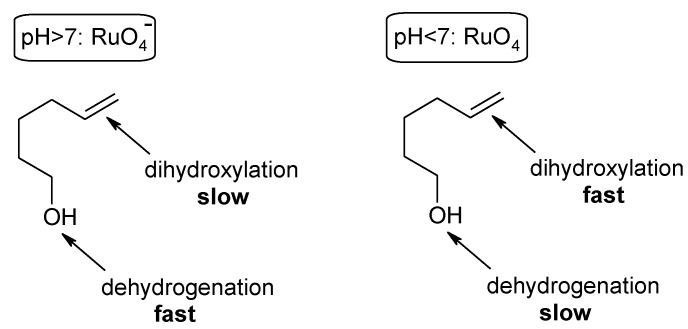
Chemoselectivity switch in the Ru-catalyzed oxidations by changing the pH [84].

**Scheme 9:**
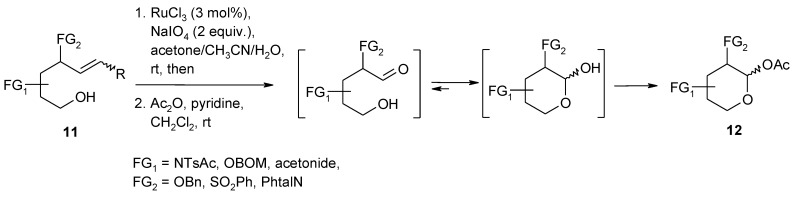
Oxidative C=C bond cleavage/cyclization/acetylation sequence [84].

**Scheme 10:**
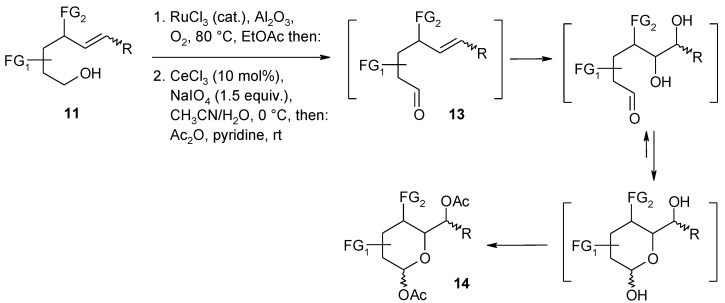
Dehydrogenation/dihydroxylation/cyclization/acetylation sequence [84,85].

A notable application of the RuO_4_-catalyzed dihydroxylation of alkenes has been reported by Plietker and co-workers [84]. In particular, a synthetic strategy was developed that allowed to obtain a small carbohydrate library based on the pH dependence of three Ru-catalyzed processes: the dehydrogenation of alcohols, the oxidative cleavage of olefins and the dihydroxylation of olefins (Scheme 8, Scheme 9 and Scheme 10). By suitably tuning the pH value of the medium, substituted δ-hydroxyalkenes **11** could be driven towards to two different oxidative routes: an oxidative C=C fragmentation/cyclization sequence (Scheme 9) or a dehydrogenation/dihydroxylation/cyclization sequence (Scheme 10). Under slightly acidic pH conditions (pH = 4–6) the stable species is RuO_4_ that causes the fragmentation of the C=C bond and the resulting aldehydes spontaneously cyclize to lactols that are eventually acetylated to give compounds **12** (Scheme 9). A slightly modified Yang’s protocol [37] was used for this transformation. Under these conditions the alcohol oxidation is slow and this functionality survives and is trapped into the lactol. On the other hand, under slightly basic conditions the stable species is the perruthenate ion that selectively oxidize the alcohol function in the same starting materials **11** to give the unsaturated aldehydes **13** (Scheme 10). Acidification of this reaction mixture to pH < 4 *in situ* generates RuO_4_ from RuO_4_^−^, which dihydroxylates the olefin function and eventually gives the undegraded acetylated lactols **14**, after acetylation.

Although the RuCl_3_/NaBrO_3_ system at pH 10 proved successful to oxidize the alcohol function in the latter sequence, it was found that the protocol developed by Yamaguchi and Mizuno [85], using molecular oxygen and the supported ruthenium catalyst RuCl_3_/Al_2_O_3_, in ethyl acetate at 80 °C, was the best method (Scheme 10).

**Scheme 11:**
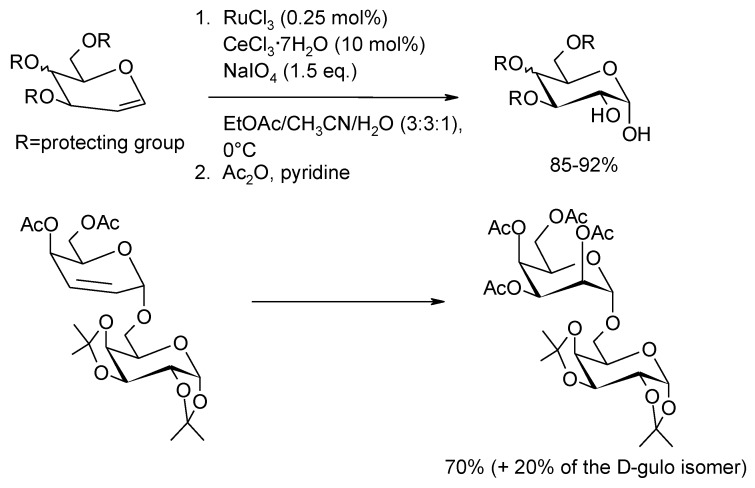
Stereoselective dihydroxylation of glycals and a 2,3-unsaturated disaccharide [86].

The dihydroxylation protocol developed by Plietker and co-workers [51] has been successfully applied in various instances. For example, the dihydroxylation of glycals and 2,3-unsaturated mono- and disaccharides [86] (Scheme 11) gave sugar 1,2- or 2,3-diols, respectively, in excellent yields and stereoselectivity. Common protecting groups used in the protection of carbohydrates such as acetyl, benzoyl, pivaloyl, TBDPS, benzylidene and isopropylidene, and even the potentially RuO_4_-oxidable benzyl group, are stable under the reaction conditions. The stereoselectivity observed is explained in all cases by the *syn* attack of RuO_4_ from the less hindered face of the olefin.

**Scheme 12:**
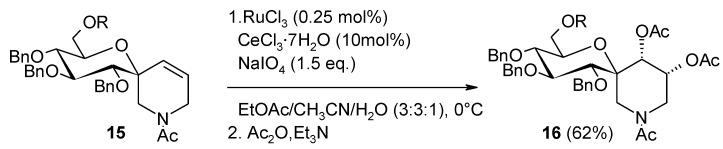
Synthesis of a glucose-tethered isofagomine analogue [87].

Another application of the RuO_4_-catalyzed dihydroxylation is found in the synthesis of a fused oxa-aza spiro sugar, a glucose-tethered isofagomine analogue designed for glycosidases inhibition activity studies [87]. Diastereoselective dihydroxylation of spiro olefin **15** (Scheme 12) failed under various OsO_4_-catalyzed dihydroxylation conditions as well as using *m*-CPBA and H_2_O_2_/HCO_2_H systems whereas the desired product **16** was obtained in 62% yield by the Ru-mediated process and acetylation.

**Scheme 13:**
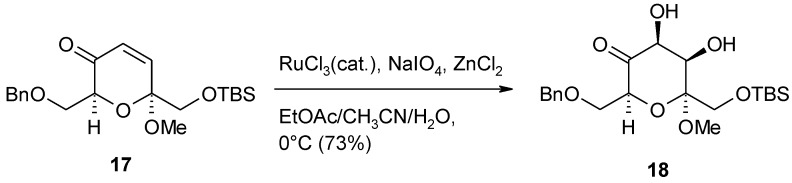
Dihydroxylation of an enone intermediate in the synthesis of the C94-C104 fragment of symbiodinolide [88].

During the synthesis of the C94-C104 fragment of symbiodinolide, a marine natural product [88], the dihydroxylation of the electron-deficient enone **17** (Scheme 13) was required. While the OsO_4_^−^ dihydroxylation procedure failed, the desired product **18** was obtained in good yields (73%) with the RuCl_3_/NaIO_4_ system in the presence of ZnCl_2_ as Lewis acid. Interestingly, the process conducted under Plietker’s standard conditions, using CeCl_3_, gave lower yields (51%).

**Scheme 14:**
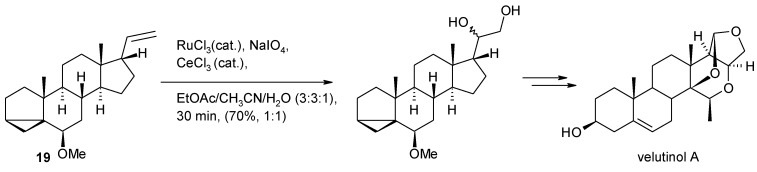
A Ru-catalyzed dihydroxylation step during the synthesis of velutinol A [89].

The synthesis of the bradykidin B1 receptor antagonist velutinol A [89] (Scheme 14), a natural product isolated from *Mandevilla velutina*, required a dihydroxylation step of the side-chain double bond in the intermediate 19. This was accomplished under Plietker’s conditions in 70% yield in short times (30 min). A similar process carried out with OsO_4_/NMO, though giving the product in higher yields (82%), required 1 day.

**Scheme 15:**
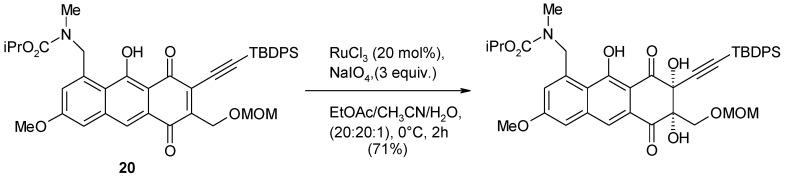
A dihydroxylation step during the synthesis of lactonamycin and lactonamycin Z [90].

The preparation of an advanced intermediate in the total synthesis of lactonamycin and lactonamycin Z [90] required the dihydroxylation of the electron-poor C–C double bond in antraquinone **20** (Scheme 15). This was accomplished under Shing’s [48] modified conditions in good yields. Notably, the potentially reactive alkyne functionality was unaffected probably as a consequence of its shielding caused by the TBDPS protecting group.

**Scheme 16:**
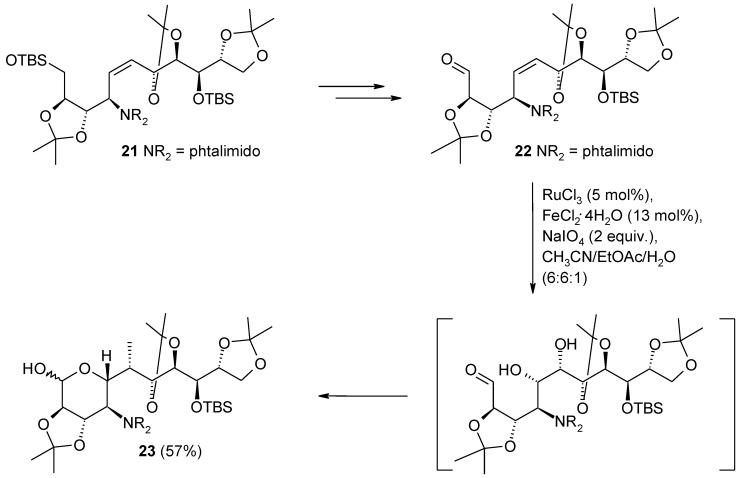
A highly diastereoselective dihydroxylation step during the synthesis of the core of hikizimycin [91].

During the synthesis of the hikosamine core of the antibiotic hikizimycin [91], the transformation of compound **21** into the hemiacetal **23** was required (Scheme 16). However, the the crucial double bond dihydroxylation in **21**, with catalytic OsO_4_, failed to give the desired diol (<5% conversion). A good conversion required the unattractive use of stoichiometric amounts of OsO_4_ in the presence of TMEDA and successive hydrolysis of the osmate ester. In addition, successive synthetic steps carried out on the dihydroxylation product were equally difficult. The problem was overcome by the RuO_4_-catalyzed dihydroxylation of the aldehyde **22**, where FeCl_2_•4H_2_O turned out to be the best Lewis acid for the conversion. Under these conditions the first-formed diol spontaneously cyclized to the required pyranose ring of **23**. Notably, the aldehyde function in **22** survived to the dihydroxylation conditions.

**Scheme 17:**
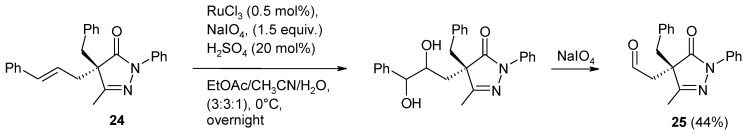
Oxidation of a pyrazol-5-one [92].

An application of the Ru-catalyzed protocol using catalytic amounts of a Bronstedt acid [50] is found in the dihydroxylation of pyrazol-5-one **24** [92] (Scheme 17) which gave aldehyde **25** in 44% yield after diol cleavage. 

**Scheme 18:**
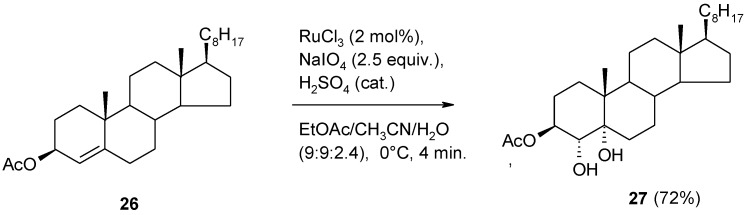
Dihydroxylation of cholest-4-en-3β-yl acetate [93].

In a recent synthesis of 4α-hydroxy-7-dehydrocholesterol by Ilida and co-workers [93] the stereoselective dihydroxylation of the Δ^4^ double bond of cholest-4-en-3β-yl acetate **26** was required (Scheme 18). While OsO_4_ and KMnO_4_ failed to give the desired product, the RuO_4_-catalyzed process was successful giving the 4α,5α-diol in a 72% yield and in a fast way. This compound had previously been synthesized in our group (Scheme 1) [43] using stoichiometric amounts of RuO_4_ in CCl_4_. Interestingly, though the experimental conditions used by the Japanese group differed, the yield of **27** was in perfect agreement with that reported by us. This is a further confirmation of the importance of the RuO_4_-mediated dihydroxylation as a valuable alternative to other methods.

### 2.4. The Ruthenium Diester Intermediates in the Oxidation of Alkenes

**Scheme 19:**
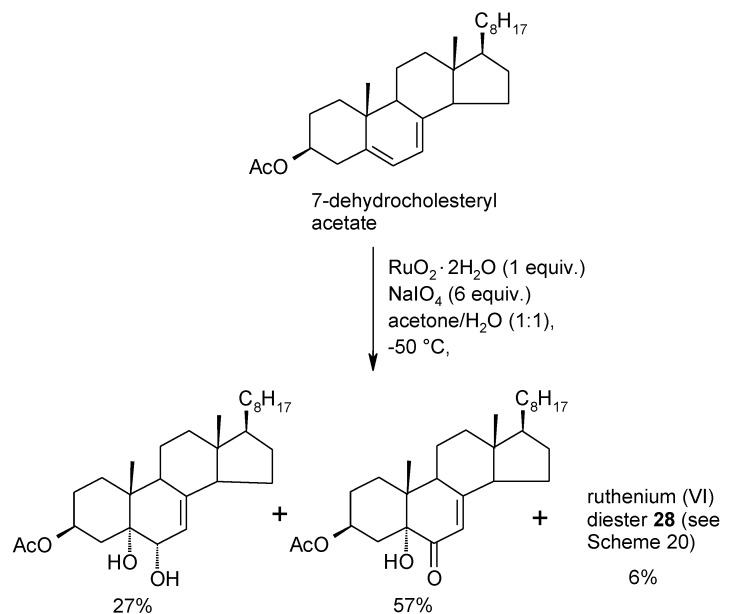
Oxidation of 7-dehydrocholesteryl acetate with RuO_4_[46].

**Scheme 20:**
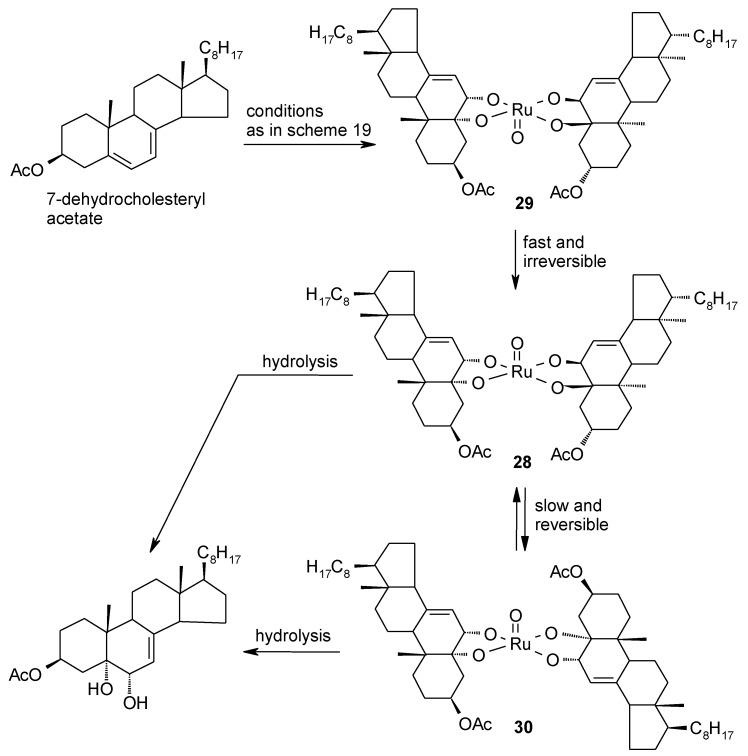
Isomeric ruthenium(VI) diesters obtained by reaction of 7-dehydrocholesteryl acetate with RuO_4_ [95].

In this section the current knowledge of ruthenate ester intermediates involved in the interaction of RuO_4_ and olefins is summarized. Kinetic studies performed by Lee and Spitzer in 1976 [94] had suggested that the RuO_4_-mediated cleavage of carbon-carbon double bonds could proceed through the formation of a cyclic ruthenium (VI) diester intermediate similar to that involved in the reaction of alkenes with OsO_4_. However, it was not until 1994 that the first ruthenium(VI) diester intermediate in the dihydroxylation of an alkene could be isolated [46]. In particular, Piccialli and Sica carried out the oxidation of 7-dehydrocholesteryl acetate with stoichiometric amounts of RuO_4_ at −50 °C, in acetone water (1:1). The reaction led to a mixture of the 1,2-diol and the corresponding α-ketol (Scheme 19) along with a precipitate that was then disclosed to be the ruthenium(VI) diester **28** (Scheme 20), analogous to the osmate ester intermediate of the second cycle of the osmium tetroxide-catalyzed asymmetric dihydroxylation of olefins [83]. A more in-depth look to this process seems appropriate here since no other example of this type of substance has been reported ever since and previous discussions of this topic were rather incomplete.

The use of stoichiometic amounts of RuO_4_ as well as both the nature of the substrate and the solvent mixture employed, all played a key role for the isolation of this substance. Indeed, the steroid substrate was soluble in the polar solvent mixture used for this reaction but when two steroidal units bonded to RuO_4_, a rather insoluble species resulted. Hydrolytic, or oxidative, decomposition of this species took place for the most part though a little amount (6%) of it precipitated subtracting itself to the further decomposition. Compound **28** proved sufficiently stable to be isolated and purified by HPLC in hexane–EtOAc mixtures and its NMR characterization could be accomplished in CDCl_3_ solutions.

In a successive study, [95] a careful investigation of this process was carried out and further evidence was gained. In particular, it was observed that three distinct species (**28**–**30**, Scheme 20) were produced during the oxidation of 7-dehydrocholesteryl acetate and that the most abundant one (>95%) was the new compound **29**. This species underwent isomerization to two other isomeric ruthenium (VI) species, namely previously isolated **28**, and the new species **30**, under mild acidic conditions (SiO_2_/CDCl_3_). The isomerization process could be monitored by ^1^H-NMR starting from pure samples (HPLC) of the three isomers and the picture shown in Scheme 20 resulted. Eventually, after several hours the mixture of **28** and **30** hydrolysed to the sole 1,2-diol product. In addition, chromatographic (TLC), and ^1^H- and ^13^C-NMR features of **28** and **30** proved strictly similar to those displayed by their analogous osmium (VI) diesters synthesized for comparison. These osmate esters showed no tendency to convert one another a fact that testimonies the different stability of these related species. In addition, no osmate ester corresponding to **29** was obtained when 7-dehydrochoelsteryl acetate was reacted with OsO_4_.

The behaviour of cholesteryl acetate was then studied under the same oxidative conditions [95]. A similar precipitate was obtained though in a smaller 1% amount. NMR evidence clearly indicated that it was composed of a 1:4 mixture of ruthenium diesters similar to **29** (major isomer) and **28** (minor isomer). The most abundant compound could be purified by HPLC and studied in pure form. It displayed a more pronounced instability compared with its analogue **29** derived from 7-dehydrocholesteryl acetate. Presumably, this can be attributed to the Δ^7^ double bond present in **29** that for some reason may play a stabilizing role. Further studies on this point, aimed at the comprehension of the stability and properties of these materials, would be suitable. As previously pointed out [72], we believe that the labile nature of the ruthenate esters of the above type, or the first-formed 1:1 species derived by cycloaddition of RuO_4_ and one molecule of the alkene, may be responsible for some of the reactions peculiar to RuO_4_ that do not have counterpart with OsO_4_ such as formation of variable amounts of *trans*-THF in the oxidative cyclization of 1,5-dienes [52], formation of *trans*-THP from 1,6-dienes [54] and the oxidative polycyclization of polyenes [57,58,59,60] (for a detailed discussion on this point see reference [72]).

**Scheme 21:**
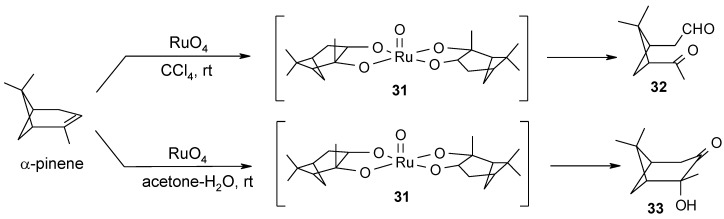
Oxidation of α-pinene with RuO_4_ [69].

In a related study, conducted in our group, NMR evidence was collected on the involvement of a similar cyclic ruthenium diester species **31** (Scheme 21), both in the oxidative cleavage and the ketohydroxylation of α-pinene [69]. Interestingly, once again the change in the solvent used for the reaction channelled the process towards two different oxidative routes. Cleavage of the olefin was observed in CCl_4_ solutions leading to ketoaldehyde **32** as the sole product whereas ketol **33** was obtained in acetone-water (2:1). NMR data collected for both processes monitored by ^1^H-NMR indicated that the same intermediate was involved but, in this case, it was too unstable and could not be isolated. However, it could be seen to migrate without decomposition on silica TLC in hexane-acetate mixtures and had the very same R_f_ value of the corresponding, stable, osmate ester, the structure of which was secured by X-ray analysis. Once again, as observed for the ruthenium(VI) diesters of 7-dehydrocholesteryl acetate, the ruthenium and osmium diesters of α-pinene showed strictly similar ^1^H-NMR features as well.

As far as this author knows, no other experimental evidence of the intermediate of the oxidation of alkenes with RuO_4_ have appeared in the literature successive to these studies, but theoretical studies were independently conducted by Strassner [96] and Frenking [97]. In both cases a [3+2] mechanism was shown to be favourite over the [2+2] mechanism and the former seems to have been definitively accepted as the operating route.

**Scheme 22:**
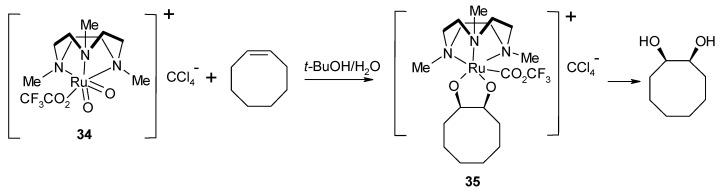
Stoichiometric cyclooctene dihydroxylation by [(Me_3_tacn)(CF_3_CO_2_)Ru^VI^O_2_]ClO_4_ [98].

Related Ru(III) species, supposed to be the intermediates of novel Ru-catalyzed alkene *cis*-dihydroxylation and alkene cleavage protocols were more recently isolated when using the *cis*-dioxoruthenium (VI) complex **34** (Scheme 22) as the dihydroxylating species [98]. The collected evidence indicated that both processes involve the direct interaction of the two oxo ligands of Ru with the C=C bond, with formation of [3+2] cyloadducts. The cycloadducts with cyclooctene (Scheme 22) and *trans*-β-methylstirene were isolated and characterized by X-ray crystallography. Though in this case no RuO_4_ is involved, once again a cycloaddition mechanism similar to that working for RuO_4_ seems to be operative.

### 2.5. Oxidative Cyclization of Dienes and Polyenes

The oxidative cyclization of 1,5-dienes to THF diols can be carried out by four metal oxo-species namely RuO_4_, RuO_4_^−^, MnO_4_^−^ and OsO_4_, with various degrees of effectiveness. The analogous cyclization of 1,6-dienes to THP diols can be carried out with RuO_4_ [54] and permanganate [99]. Interestingly, in the first case *trans*-THP are obtained in moderate to good yields. On the contrary, permanganate leads to *cis*-THP though yields not exceed 35% and a few examples have been reported. A similar transformation is not known to occur with OsO_4_. The oxidative cyclization of 1,7-dienes to *trans*-oxepanes [55,56] is peculiar to ruthenium tetroxide and a similar process is not known for other oxo-species. The RuO_4_-catalyzed oxidative mono-cyclization of 1,n-dienes has previously been reviewed [72]. Recent developments in this fields, mostly concerning the OsO_4_ chemistry, have been summarized in a review by Pilgrim and Donohoe [100] where a comparative picture of the oxidative cyclizations of 1,5- and 1,6-dienes, and related processes mediated by various metal oxo-species, including RuO_4_, has been presented. 

Kirchner *et al.* [101] carried out density functional theory studies to investigate the mechanism of the RuO_4_-mediated oxidative cyclization of 1,5- and 1,6-dienes. Considering an operating [3+2] cycloaddition mechanism, they concluded that the transition structure for the formation of the *cis*-THF ring was more stable by about 40 kJ/mol than the *trans*-THF-forming transition structure. In the formation of tetrahydropyran compounds from 1,6-dienes, a transition structure for the formation of the *trans*-THP was less that 4 kJ/mol more stable than the transition structure leading to the *cis*-THP isomer. Their conclusions were in agreement with observed experimental selectivities.

**Scheme 23:**
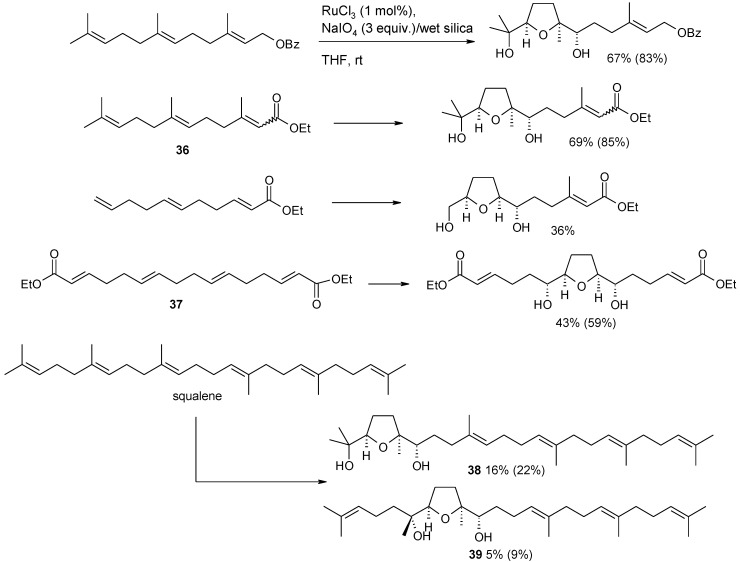
Oxidative monocyclization of polyenes. Overall yields including overoxidation products are shown in parentheses [102].

As a more recent contribution in this field Stark and co-workers investigated the mono-cyclization of a range of polyenes, namely 1,5,9-trienes, 1,5,9,12-tetraenes and squalene [102]. Selected examples are reported in Scheme 23. Various protected farnesol-derived substrates were subjected to the protocol using catalytic RuCl_3_ and NaIO_4_ on wet silica, to give mono-THF diol products in moderate to good yields. Yields are generally good if calculated including the overoxidized ketol products. The higher yield of the THF diol derived from triene 36 could be attributed to the survival of the terminal electron-deficient double bond. Non-terpenoid 1,5,9-trienes gave lower yields (21%–42%) of similar mono-THFs. Tetraenes gave mono-THF products in a 19%–51% yield. The yield and the position selectivity depends on the electronic properties of the substrate (see for example the oxidation of **37**) and possibly is influenced by steric factors. Squalene led to poor yields of the two isomeric mono-THF compounds **38** and **39** possibly due to the growing side reactions triggered by the presence of six double bonds in this compound.

**Scheme 24:**
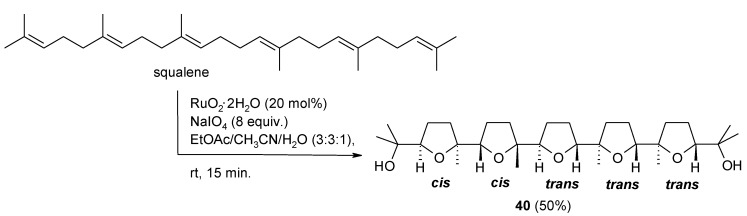
Stereoselective oxidative polycyclization of squalene with RuO_4_ under catalytic conditions [57,103].

The RuO_4_-catalyzed oxidative polycyclization (OP) of polyenes characterized by a repetitive 1,5-diene structural motif is a stereoselective cascade process discovered some years ago in our laboratories [57,58,59,60,72]. Some representative isoprenoid polyenes such as farnesyl acetate, geranylgeranyl acetate and squalene, as well as unbranched linear polyenes, were transformed into bis- tris and penta-THF products by this process. The oxidation of squalene [57] is a notable example since it is transformed into the structurally complex penta-THF compound **40** (Scheme 24) in a remarkable 50% yield in a straightforward and fast way. The process has been scaled up and multigram amounts of the product could be obtained [103].

**Scheme 25:**
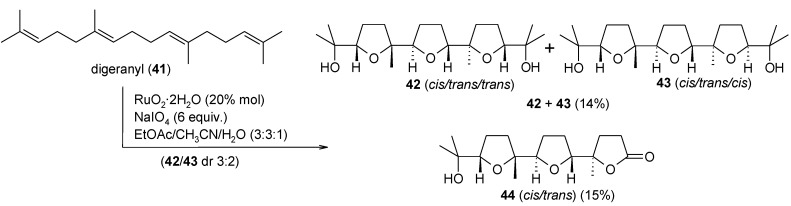
Oxidative polycyclization of digeranyl with catalytic RuO_4_ [104].

More recently we tested the reactivity of the *C_S_*-symmetric tetraene digeranyl **41** (Scheme 25) [104]. We were interested to collect further evidence on the steroselectivity of the process and in particular to evaluate the effect of the tail-to-tail fusion of the central isoprene units in digeranyl, on the second cyclization step. The oxidation of **41** led to the two isomeric tris-THF products **42** and **43** along with the stereostructurally related truncated bis-THF lactone **44** in a 29% overall yield. Interestingly, an unexpected stereochemical outcome was obtained as compared to the OP of all the other polyenes previously tested. While in previous instances the second cyclization step constantly led to a *cis*-THF ring, in this case a *trans*-THF ring was obtained in the second cyclization for all three compounds **42**–**44**.

**Figure 2:**
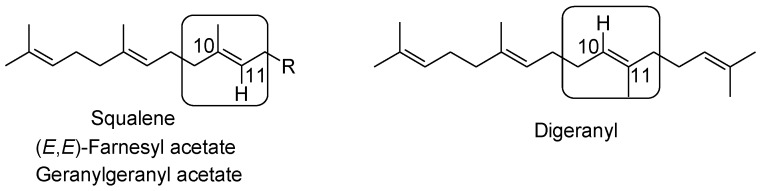
Head-to-tail type isoprenoid polyenes and digeranyl compared [104].

It is to be noted that from a structural point of view, the exchange of the Me-10 and H-11 occurs on going from FA, GGA and squalene to digeranyl (Figure 2). This structural change likely triggers the formation of a *trans*-THF in the second cyclization step.

**Figure 3:**
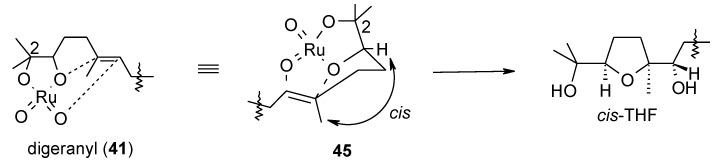
Model for the *cis*-selective first cyclization of digeranyl [52,53,60,61,106,107,108,109,110,111].

**Figure 4:**
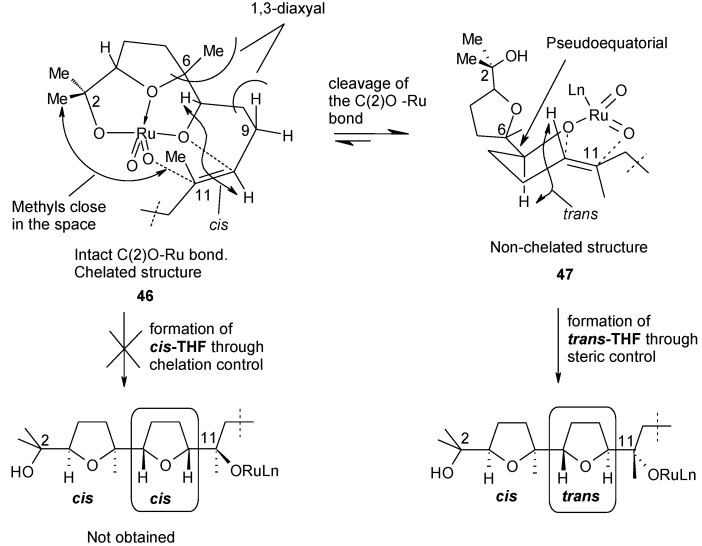
Chelation *versus* steric control in the second cyclization step of digeranyl [104].

An explanation of the observed stereoselectivity, based on previously proposed chelation/steric control models [60] operating in each cyclization step, is reported in Figure 3 and Figure 4. In particular, the *cis*-selectivity of the first cyclization step can be explained through a [3+2] cycloaddition in the intact ruthenium bis-glycolate intermediate **45** (Figure 3) with the molecule assuming a chair-like conformation in the transition state. This type of *cis*-selective cyclization characterizes the oxidative cyclization of 1,5-dienes with permanganate [105,106], OsO_4_ [107,108,109,110], RuO_4_ [19,52,53,61] and perruthenate [111].

When referring to the transition state for the second cyclization step, the conformation **46** (Figure 4), leading to the closure of a *cis*-THF ring in the second cyclization step, is disfavoured due to steric interactions within the C(11)-Me/C(2)-Me and H_ax_-9/C(6)-grouping pairs. As a result, the alternative non-chelated arrangement **47**, generated by cleavage of the C(2)O-Ru bond, is favoured leading to a *trans*-THF, through steric control.

The scarce stereoselectivity observed for the third cyclization step can be explained in a similar manner considering that the *trans* configuration of the second THF ring affects the course of the third cyclization. In particular, less severe steric repulsions in the TS for the third cyclization step result that render the *cis*-THF-forming step now more viable if compared to the analogous cyclization step for GGA and squalene and, as a consequence, both *cis*- and *trans*-THF rings can now be formed. On the other hand, formation of lactone **44**, not observed previously, indicates that the hydrolysis of the ruthenate ester intermediate in part takes place after the second cyclization step thus stopping the process. Successive oxidation/cyclization events lead to the lactone formation.

An interesting point emerged from this study: the stereoselectivity of the second and third cyclization steps in the OP of digeranyl are affected by its alkyl substitution pattern that drives the cyclization steps successive to the first one.

#### 2.5.1. Oxidative Polycyclization of Hydroxypolyenes

**Scheme 26:**
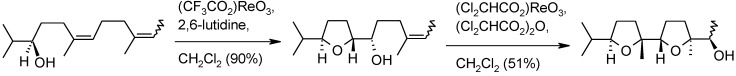
Acylperrhenate-induced, tandem *syn*-oxidative cyclization of an hydroxydiene [112,118,119,120].

**Scheme 27:**
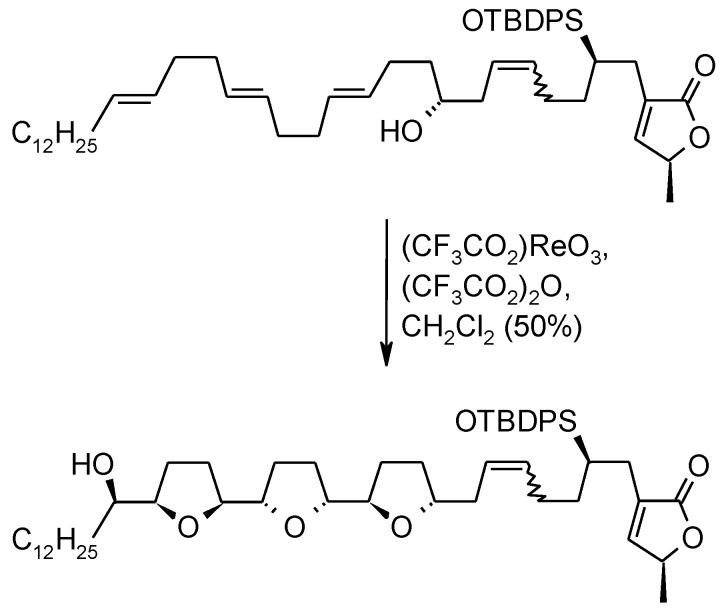
Triple oxidative cyclization of a bis-homoallylic trienol [112,121].

The above Ru-catalyzed oxidative polycyclization process is clearly related to the oxidative polycyclization of hydroxypolyenes catalyzed by CF_3_CO_2_ReO_3_ [112,113,114] that allows formation of bis- or tris- THF products in a single step. Although the closure of up to three adjacent THF rings in hydroxypolyenes has generally been accomplished in a sequential manner [115,116,117], a single-step bis-cyclization (Scheme 26) [112,118,119,120] or tris-cyclization (Scheme 27) [112,121] has been carried out in some instances. This multiple cyclization process is the evolution of the rhenium (VII) methodology developed by Kennedy and Tang [122] for the synthesis of THF alcohols, starting from alkenyl alcohols. The system CF_3_CO_2_ReO_3_/(CF_3_CO_2_)_2_O, developed by McDonald and co-workers showed to be the best choice to induce the formation of two or three contiguous THF rings in a single step. This chemistry has mostly been applied to the synthesis of the poly-THF core of annonaceous acetogenin metabolites. However, no case of the Re(VII)-mediated formation of poly-tetrahydrofuran products with four or more adjacent THF rings has been reported.

**Scheme 28:**
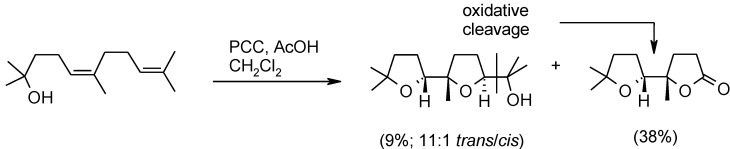
Bis-cyclized (only the *trans* isomer is shown) and cleavage products by PCC-mediated oxidative cyclization [123].

McDonald and Towne [123] also showed that pyridinium chlorocromate (PCC) could induce the *syn*-oxidative cyclization of hydroxydienes (Scheme 28). However, the method suffers by oxidative cleavage pathways and was limited to tertiary alcohols due to the rapid oxidation of primary and secondary alcohols by PCC.

It is worth highlighting some differences between the RuO_4_-catalyzed and the Re(VII)-mediated processes. While the Re(VII)-mediated process constantly gives a *trans*-THF in the first cyclization, a *cis*-THF is always obtained in the first cyclization in the Ru-catalyzed transformation (Scheme 25 and fig. 3). This is a direct consequence of the type of ester intermediate formed in the initial step. While the Re-mediated process needs an OH group in the structure to give a Re(VII) ester intermediate (RO-ReO_3_), the Ru-mediated oxidation begins with a [3+2] cycloaddition of RuO_4_ and a C–C double bond, leading to a ruthenium(VI) diester intermediate. As a consequence, a poly-tetrahydrofuran-diol product is obtained with RuO_4_, while a mono-, bis- or tris-tetrahydrofuran alcohol is obtained when a rhenium(VII) oxo-species is used.

**Scheme 29:**
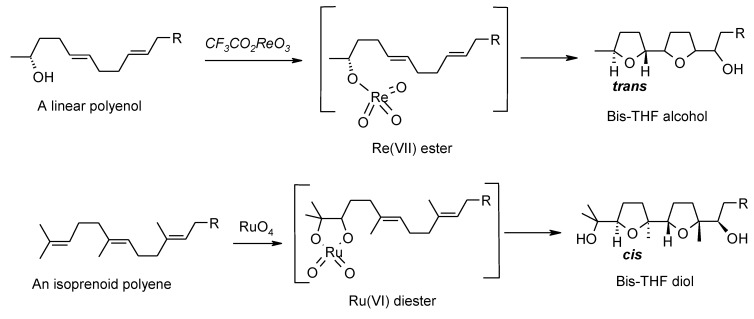
Comparison of Re- and Ru-mediated oxidative polycyclization of a polyenol and a polyene, respectively.

### 2.6. Oxidation of Ethers

**Scheme 30:**
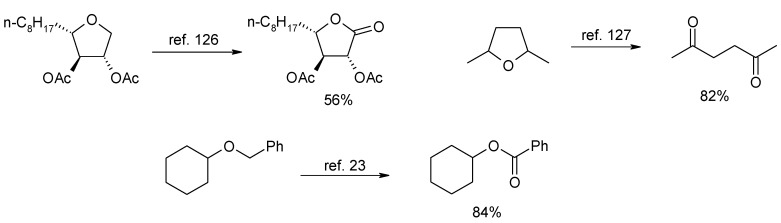
Representative examples of the RuO_4_-catalyzed oxidation of ethers.

**Scheme 31:**
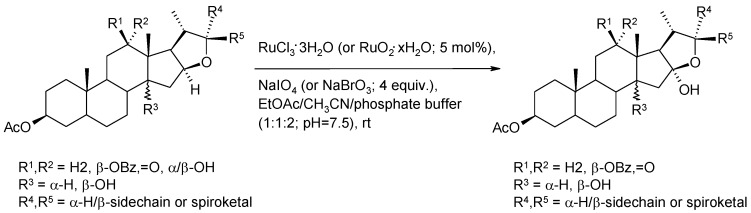
RuO_4_-catalyzed oxyfunctionalization of steroidal ethers [130].

**Scheme 32:**
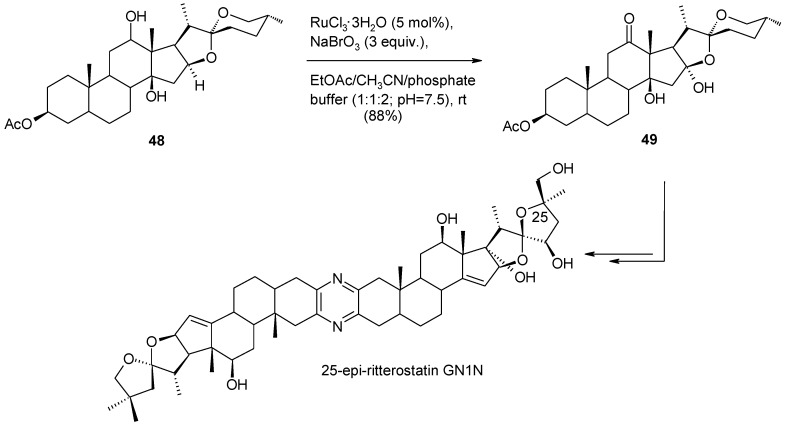
C-14 oxyfunctionalization of C-12,14 dihydroxy hecogenin acetate [131].

Ethers possessing a methyl or a methylene group adjacent to the oxygen are generally oxidized in good yields to esters by RuO_4_ (Scheme 30) [17,124,125,126]. This chemistry can be fruitfully used, for example, to convert benzyl protecting groups into benzoates (Scheme 30) [23] thus modifying the removal conditions of the former. On the contrary, few examples have been reported on the oxidation of ether methine carbons [127,128] in cyclic or acyclic ethers though the oxidative opening or the oxyfunctionalization of 2,5-disubstituted cyclic ethers would have synthetic value [129]. An interesting example of this type of transformation has been reported by Fuchs and co-workers [130] where acid-sensitive steroidal ethers were oxidized to hemiketals under new developed buffered conditions. (Scheme 31). This process has recently been used by the same group to synthesize 14,16-dihydroxyhecogenin acetate **49** in high yields (Scheme 32), a key intermediate in the synthesis of the potent anticancer cephalostatin/ritterazide hybrid 25-*epi*-ritterostatin G_N_1_N_ [131], starting from C-12,14 dihydroxy hecogenin acetate **48**. Notably, 90.5 g of compound **49** could be obtained starting from 100 g of **48**.

**Scheme 33:**
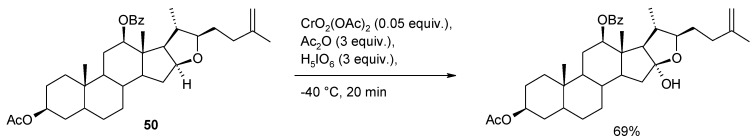
Chromyl acetate catalyzed oxyfunctionalization [132].

A strictly similar chromyl acetate-mediated oxyfunctionalization process has been reported by the same group [132]. Chromyl acetate was used in catalytic amounts in the presence of periodic acid as primary oxidant to hydroxylate the C-14 position of the steroidal substrate **50** (Scheme 33). The authors postulate the intermediacy of a chromoylperiodate species that evolves to a peroxo intermediate which then adds to the C(14)-H bond.

A related ether oxidation process is represented by the oxidative cleavage of THF and THP alcohols (β-hydroxy ethers) to γ- and δ-lactones, respectively. Some examples of this type of RuO_4_-mediated transformation have been shown above (Scheme 25; see also Scheme 39 later). While this is generally a detrimental side-process in oxidative mono- and poly-cyclizations, it may have *per se* synthetic value since lactones of various sizes are structural motifs in many natural products.

**Scheme 34:**
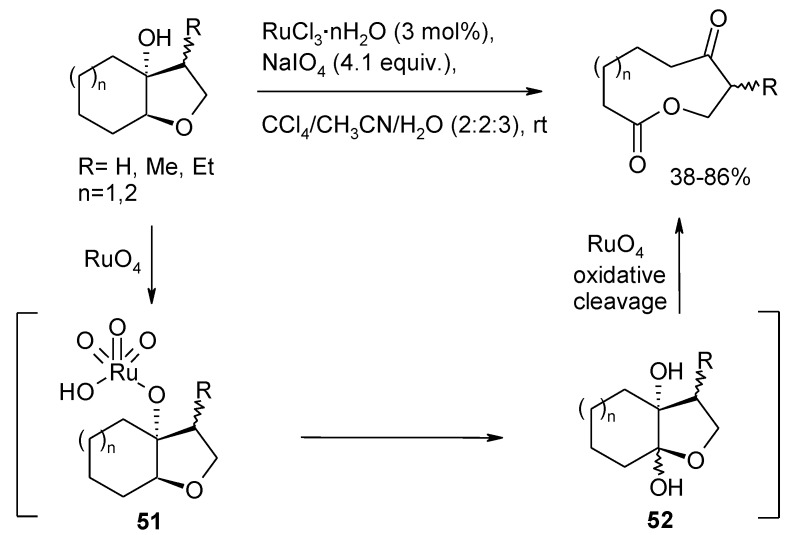
Synthesis of ketolactones by oxidative cleavage of β-hydroxytetrahydrofurans [133,134].

As a continuation of their previous studies [133], Ferraz and Longo Jr. [134] used this RuO_4_-catalyzed transformation to obtain nine and ten- membered functionalyzed lactones by oxidative cleavage of *cis*-fused bicyclic β-hydroxytetrahydrofurans (Scheme 34).

**Scheme 35:**
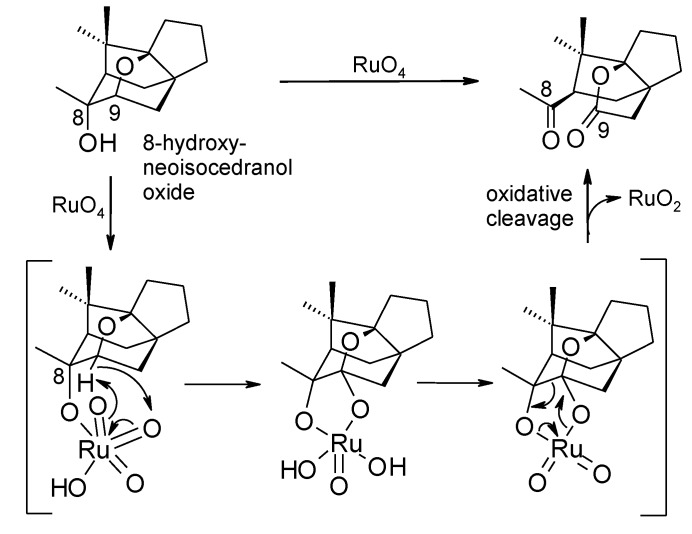
Mechanism proposed for the oxidative cleavage of 8-hydroxy-neoisocedranol oxide with RuO_4_ [135].

Since the β-positioned hydroxyl group was shown to be essential for the process, it is likely that the process involves the preliminary formation of ruthenate ester **51** (Scheme 34). The tethered oxoruthenium appendage then participates to the oxidation of the angular methyne ether. These authors presume that the oxidation generates an intermediate diol compound **52**, that is then oxidatively cleaved by RuO_4_ itself. However, a concerted step can also be envisaged were a cyclic ruthenium (VI) ester is formed that then fragments, in accord with the mechanism proposed by Waegell and co-workers for the oxidation of 8-hydroxy-neoisocedranol oxide (Scheme 35) [135]. As far as this author knows, all the reported examples of this type of process involving RuO_4_ are relevant to THF or THP rings flanked by tertiary alcohol functions.

A similar chemistry is promoted by pyridinium chlorochromate (see for example Scheme 28) [123]. Baskaran and Chandrasekaran [136] used PCC to convert a range of THF-methanol compounds to γ-lactones. In this case, the process worked well with substrates possessing primary, secondary and tertiary alcohol moieties. Substrates possessing primary or secondary hydroxyl groups gave the products in moderate yields (52–58) whereas excellent yields (73%–98%) were obtained for those embodying tertiary alcohol functions. Synthetic applications of this process have been reported [59,104,137,138,139,140,141].

**Scheme 36:**
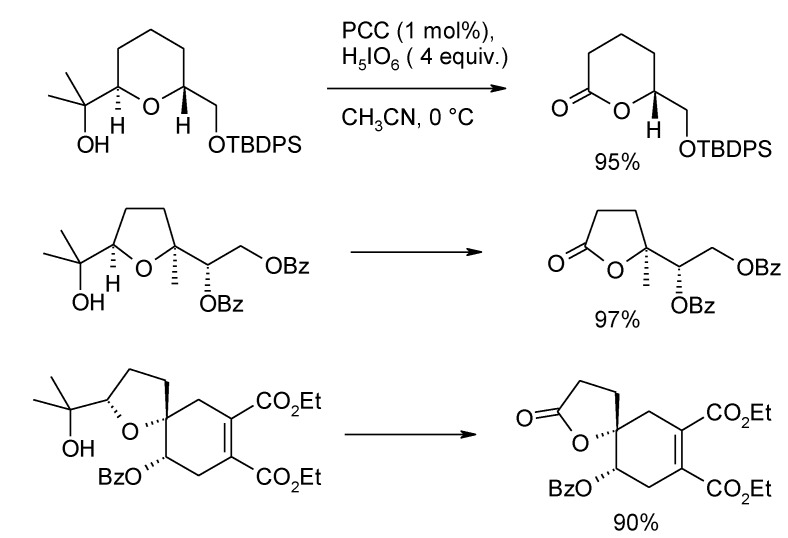
Oxidative cleavage of tertiary THF and THP alcohols with PCC/H_5_IO_6_ [142].

In a more recent investigation, Roth and Stark [142] developed an improved procedure involving catalytic amount of PCC and periodic acid as co-oxidant. THF or THP rings flanked by tertiary alcohol groups were cleaved in excellent yields (Scheme 36). It is presumed that chlorochromate is first activated by reaction with periodic acid to form a mixed anhydride [143], that is more reactive as compared to PCC, in a manner similar to that proposed for the above chromyl acetate-catalyzed process.

#### 2.6.1. Oxidative Spiroketalization

**Scheme 37:**
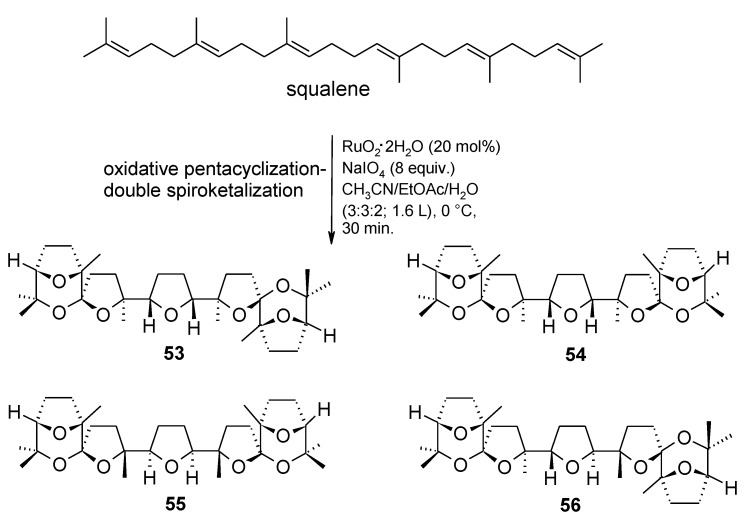
Novel C_30_ bis-spiroketals by RuO_4_-catalysed tandem oxidative polycyclization/double oxidative spiroketalization of squalene [79].

**Scheme 38:**
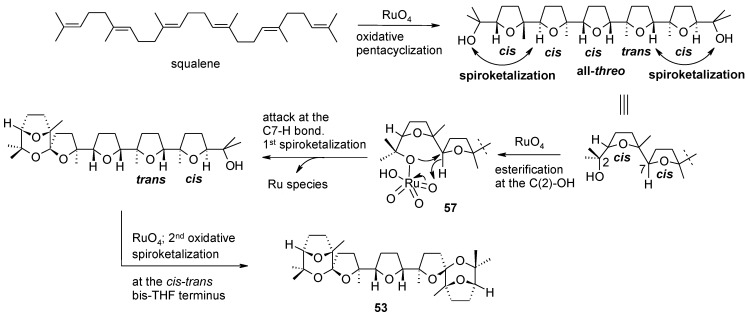
A mechanistic hypothesis explaining the formation of compound **53** [79].

In a recent study carried out in our group, a new RuO_4_-catalysed tandem oxidative polycyclization/double oxidative spiroketalization process [79] was discovered, by conducting the oxidation of squalene under new conditions. This process allowed the one-step assembly of the new structurally complex polyether bis-spiroketals **53**–**56** characterized by unprecedented terminal tricyclic spiroketal moieties (Scheme 37). Contrary to the OP of squalene under previous conditions [57,59], in this case the process was not stereoselective. Only the first THF-forming step proceeds with the usual *cis*-selectivity [52,53,57,58,59,60,61,62] whereas all the other THF rings were formed in a non-stereoselective manner with a *cis* or *trans* configuration. The reason of this behaviour is still unclear. The overall yields of these compounds was of ca. 5% but considering the seven consecutive cyclization steps involved, this is a remarkable result (65% per cyclization step) and hundred mg of these material could be obtained conducting the process on 122 mmol of squalene. Indeed this is the most complex transformation catalyzed by RuO_4_ ever discovered. A mechanistic hypothesis explaining the formation of compound **53** is shown in Scheme 38. Interestingly, compounds **53**–**56** exhibited antitumor activity on HEY ovarian-derived cancer cell line and BT474 breast-derived cancer cell line paving the way to the investigation of a new class of cytotoxic substances.

**Scheme 39:**
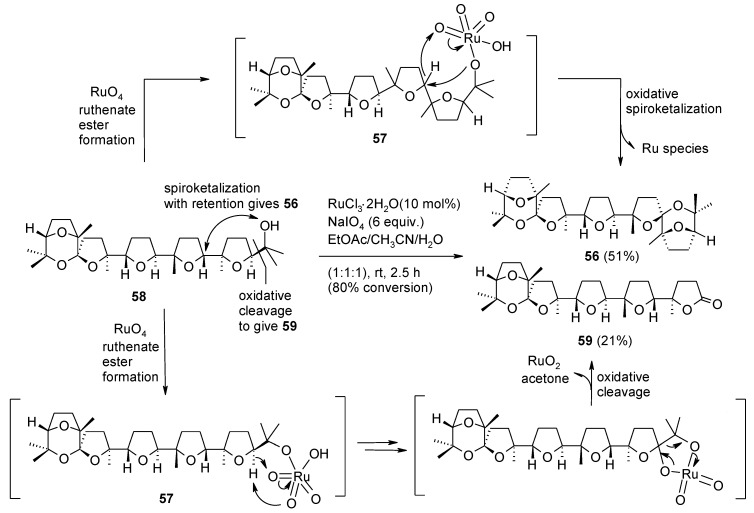
RuO_4_-mediated oxidative mono-spiroketalization and the competing oxidative cleavage [79].

To further study the spiroketal-forming step involved into the process, mono-spiroketal **58** (Scheme 39) was treated with the RuCl_3_(cat)/NaIO_4_ system under further optimised conditions. Bis-spiroketal **56** was obtained from mono-spiroketal **58** at 80% conversion along with a minor amount of lactone **59** derived from the cleavage of the terminal hydroxypropyl moiety. The two competing processes appear to involve the same ruthenium-containing intermediate **57**. Interestingly, the spiroketalization process proceeds with retention of configuration at the newly-formed spiro-centre, as observed for the hydroxylation of epicedrane at C-8 under similar conditions [135]. The oxidative cleavage step may follow the mechanism proposed by Waegell and co-workers [135]. Although more detailed studies are to be carried out to probe the scope and limitations of this transformation, it may have synthetic value.

**Scheme 40:**
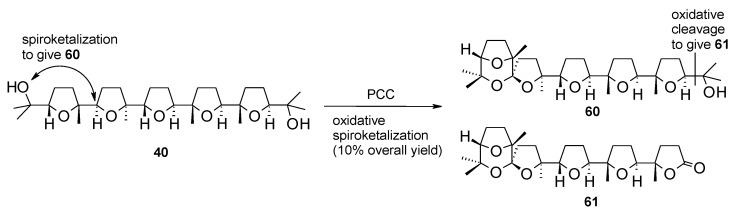
PCC-mediated oxidative spiroketalization [144].

Related studies conducted in our group showed that the same oxidative sproketalization could be accomplished by treating penta-THF **31** with pyridinium chlorochromate (Scheme 40) [144]. Compounds **60** and **61** were obtained in this case, though in a low 10% yield. The process proceeds with retention of configuration in this case too. Lactone **61** derived from the oxidative cleavage of the terminal hydroxypropyl side-chain in a manner similar to that shown above in the RuO_4_-mediated process (Scheme 39) and in the bis-cyclization of hydroxydienes with the same oxidant (Scheme 28). This reaction further highlights that RuO_4_ and PCC show similar oxidizing behaviour a fact that may have mechanistic and theoretical implications.

### 2.7. Some Unexpected Results

**Scheme 41:**
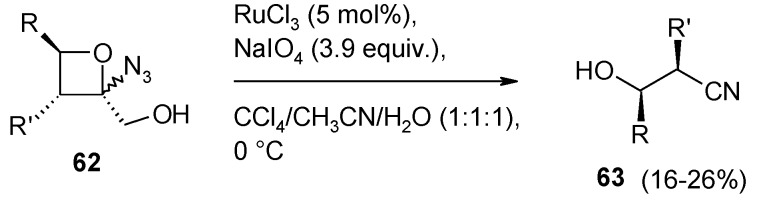
Cleavage of 2-azido-2-(hydroxymethyl)oxetanes with RuO_4_ [145].

**Scheme 42:**
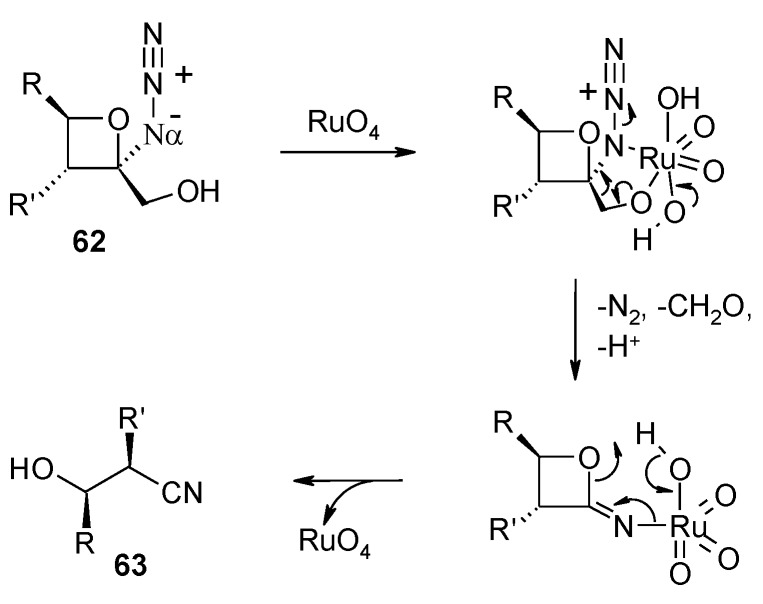
Proposed mechanism for cleavage of 2-azido-2-(hydroxymethyl)oxetanes with RuO_4_ [145].

2-Azido-2-(hydroxymethyl)oxetanes **62** were transformed into acyclic nitriles **63** with loss of the alcohol carbon (Scheme 41) by reaction with RuCl_3_ (cat.)/NaIO_4_ under classical conditions [145]. The process proceeds with low yields but is interesting from a mechanistic point of view. The authors proposed (Scheme 42) the interaction between RuO_4_ and both the alcohol hydroxyl and the N_α_ atom of the azido group. The so formed five-membered, Ru-containing, ring then cleaves with expulsion of molecular nitrogen and formaldehyde. Eventually the opening of the oxetane ring follows with nitrile formation and regeneration of RuO_4_. As far as this author knows, this is the sole known example of the interaction of RuO_4_ with the azide group. The ability of transition metals to coordinate azides [146] and cyclic 2-azidoalcohols [147] is known and the mechanism proposed seems plausible.

**Scheme 43:**
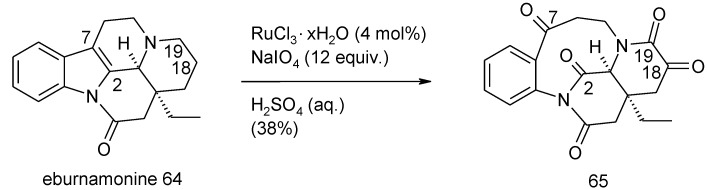
Oxidation of eburnamonine with catalytic RuO_4_ [148].

During the synthesis of melohenine B the cleavage of a tetrasubstituted double bond in eburnamonine **64** was required (Scheme 43) [148]. The catalytic system RuCl_3_/NaIO_4_, using excess periodate (12 equiv.), led to the desired C2/C7 cleavage but the concomitant oxidation of the pyperidine ring occurred with formation of an unexpected α-ketoamide functionality in the product **65**. A low number of equivalents of periodate gave a complex mixture of products. The structure of **65** was secured by X-ray analysis. While the oxidation of the carbon adjacent to nitrogen is well known [76 and references therein], this is an unusual result. The authors do not propose any mechanism for this transformation though the formation of a C-18/C-19 unsaturated intermediate, could be involved.

**Scheme 44:**
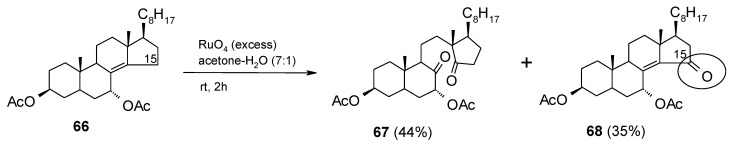
Oxidation of 5α-cholest-8(14)-ene-3β,7α-diol diacetate with RuO_4_ [43].

Another interesting result was obtained some years ago by Piccialli and co-workers [43]. In particular, the oxidation of the Δ^8(14)^ steroid **66** (Scheme 44) performed with stoichiometric amounts of RuO_4_ gave, in addition to the expected cleavage product **67**, the C-15 ketosteroid **68** derived from an unusual allylic oxidation. Similar results (C-C double bond oxidative cleavage plus a double allylic oxidation) were obtained by Rodewal and Jagodzinski for the oxidation of 3β-acetoxylanost-8-ene, 3β-acetoxy-25,26,27-trinorlanost-8-en-24-oic acid methyl ester [149], and 3β-acetoxylanost-8-en-25-ol [150] using the RuO_2_(cat.)/NaIO_4_ system.

In view of the RuO_4_ reactivity this result is difficult to be explained. It is interesting to note that the allylic oxidation occurs with sterically hindered, tetrasubstituted, Δ^8^ and Δ^8(14)^ double bonds. No other example of this type of reactivity has later been reported. However, ruthenium-catalyzed allylic oxidations of olefins to enones is a known transformation and various ruthenium reagents have been used [151]. For example, the use of ruthenium (III) chloride, in conjunction with *tert*-butyl hydroperoxide as stoichiometric oxidant, has been reported [151].

### 2.8. Oxidation of Alkanes

The oxidation of alkanes is a process of great industrial importance as well as biological relevance [152]. For the sake of completeness we will briefly mention this type of transformation here. The inherent non reactive nature of unactivated hydrocarbons makes it necessary to use strong oxidizing reagents to accomplish these oxidations and ruthenium tetroxide is one of these species. The RuO_4_-catalyzed oxidation of alkanes has mostly been independently addressed by Bakke [153,154,155,156,157,158] and Waegell [159,160,161] using the classical RuCl_3_(cat)/NaIO_4_ system. The order of reactivity in these transformations is known to be CH>CH_2_>CH_3_ and the oxidation takes preferentially place at the less hindered tertiary C-H bonds. On the other hand, the oxidation of various alkanes containing the cyclopropane ring showed that the oxidation of methylenes α to the cyclopropyl group takes place to give ketones [162]. When such a position is tertiary, and is part of a ring, cleavage of the ring occurs possibly through a hydroxylation/dehydration/cleavage sequence. Interestingly, in no case the cyclopropyl group is affected and it simply plays an activating role in these oxidations. Following the independent mechanistic proposals by Bakke and Waegell, Drees and Strassner [163] carried out theoretical studies that substantiated a [3+2] mechanism for the alkane oxidations that was in good agreement with experimental Bakke’s results.

**Scheme 45:**
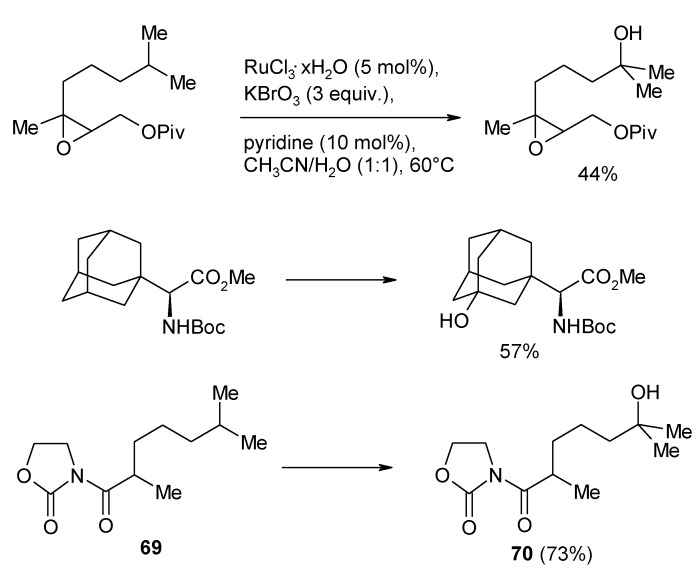
Ruthenium-catalyzed tertiary C-H bonds hydroxylation [164].

The most recent contribution on this topic is represented by the RuO_4_-catalysed selective hydroxylation of tertiary C-H bonds in a range of variously functionalised substances, developed by McNeill and Du Bois (Scheme 45) [164]. Although NaIO_4_ had previously been reported to be the best reoxidant for the alkane oxidations [135], these authors developed a new procedure employing catalytic amounts of RuCl_3_ and KBrO_3_ as primary oxidant, in the presence of catalytic pyridine. The yields of the process were generally higher than 50%, with best yields obtained by using a 1:1 mixture of H_2_O/MeCN as solvent. The new protocol was tested for the oxidation of some substrates, in comparison with the protocols previously employed by Bakke and Waegell. A substantial improvement was observed and the presence of pyridine was also shown to be crucial for the process as lower yields resulted in its absence. Functional groups such as ester, epoxide, sulfone, unprotected tertiary alcohols, carbamate and sulfamate, were tolerant to these conditions. As previously observed, when multiple tertiary positions competed for oxidation, the hydroxylation occurred at the most electron-rich C-H bond that is at the site as remote as possible from the electron-withdrawing group. For example, a >20:1 ratio of isomeric alcohols, in favour of **70**, was obtained on oxidation of **69**.

**Scheme 46:**
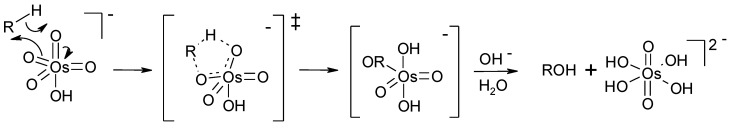
Proposed mechanism for the osmium tetroxide oxidation of alkanes [165].

More recently, Mayer and co-workers have shown that osmium tetroxide can oxidize alkanes both under stoichiometric and catalytic conditions, using periodate as co-oxidant [165]. The oxidation of a small number of alkanes was conducted at 85 °C and pH = 12.1 for 7 days. Isobutane gave *tert*-butyl alcohol, cyclopentane gave glutarate, cyclohexane gave a mixture of adipate and succinate, *cis*-decalin gave *cis*-9-decalinol. Although a radical mechanism could not be firmly ruled out [166], the collected data were in agreement with a concerted [3+2] mechanism (Scheme 46) similar to that proposed for related RuO_4_ alkane oxidations and the proposed mechanism for H_2_ oxidation by OsO_4_ [167] in the presence of hydroxide. The reactive species is thought to be OsO_4_(OH)^−^.

Permanganate [168] and references therein] and oxochromium (VI) reagents such as chromyl acetate and chromyl chloride [169,170,171,172] are well-known to oxidize alkanes. The above examples and literature data indicate that the RuO_4_-catalyzed process has the greatest synthetic potential both for regioselectivity and effectiveness reasons.

## 3. Perruthenate Chemistry

### 3.1. An Overview of the Previous Work

Tetrapropylammonium perruthenate was introduced by Griffith and Ley in 1987 [173]. Since then it has been used in catalytic amounts, in combination with *N*-methylmorpholine *N*-oxide (NMO) as co-oxidant, for the mild oxidation of a wide range of primary and secondary alcohols to carbonyl compounds [174,175,176]. Many functional and protecting groups are unaffected under these conditions. More recently, NaOCl has been employed as co-oxidant [177,178] for the oxidation of secondary alcohols under biphasic (methyl *tert*-butyl ether/water or EtOAc/water mixtures) and buffered conditions (pH 9.5). The TPAP/O_2_ pair has also been used [75,179,180,181] as a green oxidizing system and many efforts have been directed towards the development of effective aerobic oxidations. A polymer-supported perruthenate (PSP) system was developed by Ley and co-workers [181,182] and used to oxidize alcohols using oxygen as a stoichiometric oxidant [183]. However, it was later shown that this PSP reagent was difficult to recycle and other supports were investigated by the same group. These studies led to develop a more efficient heterogeneous TPAP-based catalyst [184] by immobilizing perruthenate on the internal surface of MCM-41, a mesoporous siliceous material, that was once again used to cleanly oxidize alcohols with molecular oxygen. Similarly, Pagliaro and Ciriminna prepared an organic modified silica, referred to as ormosil, doped with TPAP, through the sol-gel process, that was used for the aerobic oxidation [185,186] of alcohols or using H_2_O_2_ [187]. The same group [188] and others [189] developed aerobic alcohol oxidation procedures in supercritical CO_2_ by using silica-supported ionic liquid doped with perruthenate. Recently, some aspects of the perruthenate chemistry, including PSP chemistry, has been reviewed by Pagliaro and co-workers [75,190].

The TPAP/NMO system has also been used to oxidize functional groups other than alcohols. Thus, secondary amines were oxidized to imines [191], *N,N*-disubstituted hydroxylamines to nitrones [192], 1,4-dihydropyridines to pyridines [193], 1,2,4-triazenes to triazoles [194], while pyrrolidinones could by obtained starting from aminoalcohols [195]. A protocol for the direct conversion of alkenes to carbonyl compounds *via* alkylborane intermediates was also developed [196]. TPAP was also used as a catalyst in an efficient isomerization of allylic alcohols into the corresponding saturated carbonyl compounds [197], in the presence of 2-undecanol as a sacrificial alcohol additive. In this case it was postulated that a Ru(III) species is generated from perruthenate by oxidation of the saturated alcohol, that then enters the catalytic cycle. In the present account we will essentially focus on some transformations, recently appeared in the literature, that lends themselves to interesting synthetic applications.

### 3.2. Formation of THF-Diols from 5,6-Dihydroxyalkenes

**Scheme 47:**
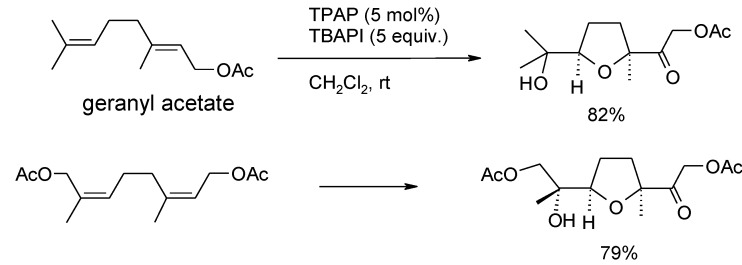
Selected examples of TPAP-catalyzed oxidative cyclization of 1,5-dienes [111].

Although double bonds and polyenes are normally unaffected by TPAP-catalyzed oxidations, some years ago Piccialli and Caserta observed that 1,5-dienes were stereoselectively transformed into 2,5-disubstituted *cis*-tetrahydrofurans (Scheme 47) by using catalytic amounts of TPAP in the presence of both NMO or tetrabutylammonium periodate (TBAPI) [111]. The process is similar to the oxidative cyclization of 1,5-dienes catalyzed by other metal-oxo species previously developed [72].

**Scheme 48:**
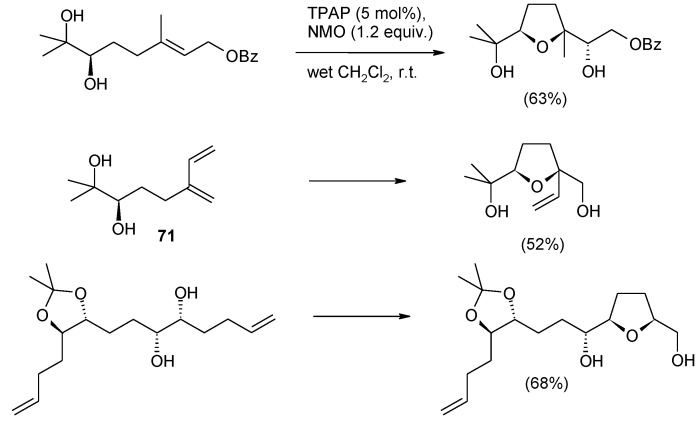
TPAP-catalyzed oxidative cyclization of 5,6-dihydroxy olefins [198].

In a related study Cheng and Stark [198] recently discovered that the TPAP(cat.)/NMO system can induce the oxidative cyclization of 5,6-dihydroxyalkenes to THF diols. Enatiomerically pure 5,6-dihydroxy olefins were cyclized to enantiomerically pure THF-diols in high diastero- and enantiopurity (d.r. >95:5; e.r. 88:12 to > 99:1) with complete stereochemical transfer. The yields were generally good and a range of substrates was screened. Some representative examples are shown in Scheme 48. Only double bonds suitably positioned to form a THF ring were reactive. In fact, cyclization of diene **71** demonstrates that formation of the THF ring is favoured over the THP ring that, in principle, could also be generated [54,63].

**Scheme 49:**
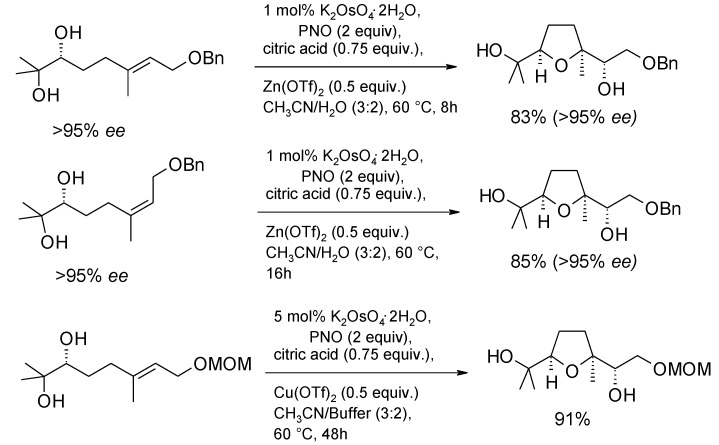
OsO_4_-catalyzed oxidative cyclization of 5,6-dihydroxy olefins under improved Donohoe’s conditions [199,200,201].

**Scheme 50:**
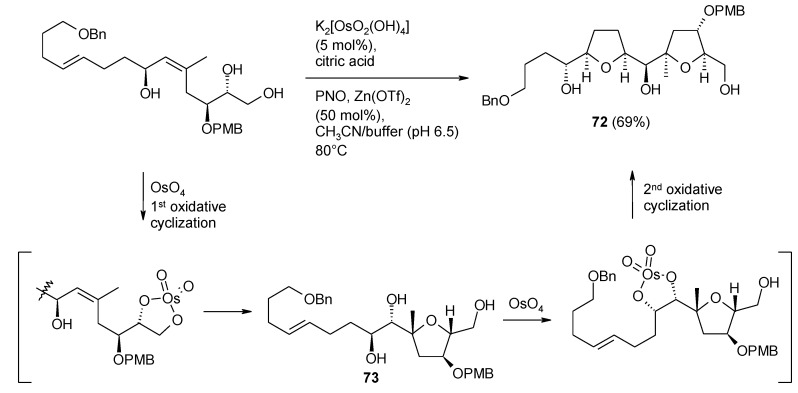
One-pot double oxidative cyclization in the synthesis of the ABC ring system of pectenotoxin-4 [202].

The above process is analogous to the osmium tetroxide-catalyzed oxidative cyclization of 5,6-dihydroxyalkenes discovered by Donohoe and Butterworth [199]. After various improvements carried out in the Donohoe’s group [200,201], this methodology has become a valuable tool for the enantioselective construction of THF rings (Scheme 49). The synthesis of various biologically active natural products, such as the annonaceous acetogenins (+)-*cis*-solamin, (+)-*cis*-sylvaticin, and (+)-sylvaticin, (−)-neodysiherbaine [100], and more recently the ABC ring system of pectenotoxin-4 [202] (Scheme 50), has been accomplished by the same group. As for this latter target, the method allowed the one-pot construction of the bis-THF synthetic intermediate **72** through a cascade oxidative cyclization step, further disclosing the synthetic relevance of this methodology and expanding its applicability. The first cyclization delivers a diol system (**73**, Scheme 50) that is appropriately placed relative to the second double bond to allow the second cyclization to take place. It is not known weather this intermediate is actually formed or the entire process proceeds with translocation of the same osmium atom on to the newly generated diol.

A similar process can be carried out with chromium trioxide and PCC. Discovered by Casida and coworkers [203] and later studied by Walba and Stoudt [204] it has rarely been used in synthesis probably due to the moderate yields (40%–50%). Corey and Ha [205] employed this process to obtain a chiral THF diol fragment to be used in the synthesis of venustatriol. 

At the moment, the osmium-catalyzed process is the most effective one to induce the cyclization of 5,6-dihydroxyalkenes.

### 3.3. Other TPAP-Catalyzed Oxidations

**Scheme 51:**
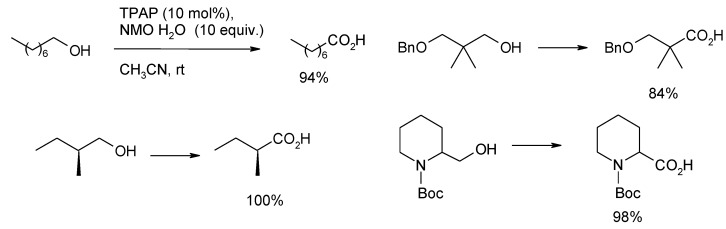
TPAP-catalyzed oxidation of primary alcohols to carboxylic acids [206].

Although TPAP is usually used to oxidize primary alcohols to aldehydes, Stark and co-workers have recently developed an efficient oxidation procedure for the direct conversion of primary alcohols into carboxylic acids using catalytic amounts of TPAP in the presence of NMO•H_2_O [206]. It was postulated that the latter plays a dual role: stabilization of the aldehyde hydrate intermediate while acting as a co-oxidant to regenerate the active Ru(VII) catalyst. For this purpose it was found that 10 equiv. of NMO•H_2_O were required. A range of primary alcohols were oxidized leading to carboxylic acids in good to excellent yields in short reaction times (Scheme 51). Several functionalities are unaffected and α and β-stereocentres remain intact as well.

**Scheme 52:**
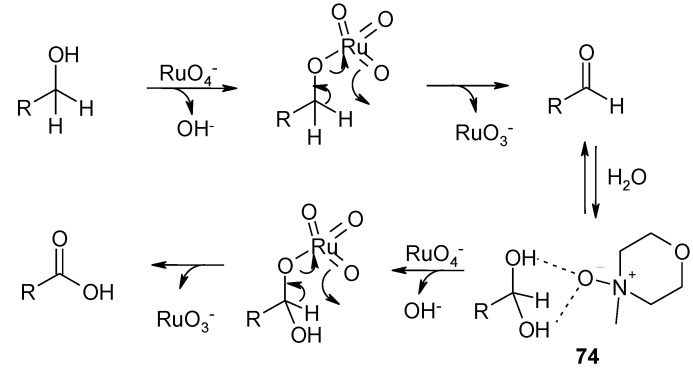
Proposed mechanism for the TPAP-catalyzed oxidation of primary alcohols to carboxylic acids [206].

The proposed mechanism is shown in Scheme 52. The oxidation to the aldehyde intermediate proceeds in the usual way [71]. The aldehyde is then hydrated and the hydrate form is stabilized by interaction with NMO to give species **74**. The latter is engaged into a Ru(VII) ester-formation step with perruthenate. Hydrogen transfer to the oxo-ruthenium moiety then follows to deliver the carboxylic acid.

**Scheme 53:**
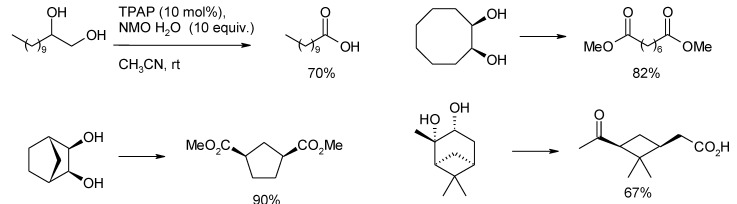
TPAP-catalyzed oxidative cleavage of 1,2-diols to carboxylic (di)acids [207].

The same oxidizing system was employed by Stark and co-workers to accomplish the direct oxidative cleavage of 1,2-diols to carboxylic acids or diacids [207]. Previously the TPAP-catalyzed oxidative cleavage of *vic*-diols to dialdehydes had been observed in some instances [208]. Since normally the above transformation requires two successive oxidizing steps (oxidative cleavage of the 1,2-diol and aldehyde oxidation), the added value of this method resides in the one-step accomplishment of the transformation. Selected examples of the new transformation are shown in Scheme 53. Some of the products were isolated as methyl esters.

**Scheme 54:**
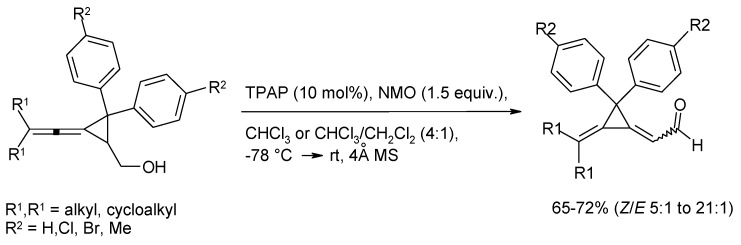
Oxidation/isomerization of vinylidenecyclopropanes with TPAP(cat.)/NMO [209].

**Scheme 55:**
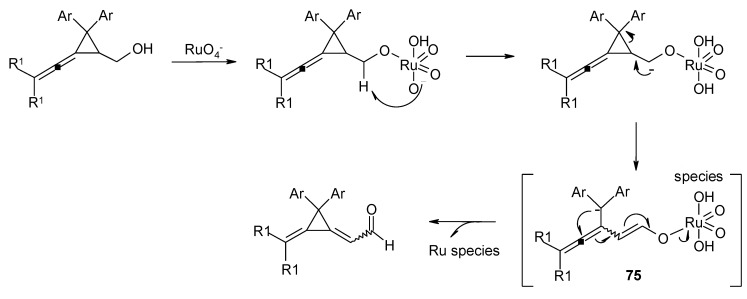
Proposed mechanism for the oxidation/isomerization of vinylidenecyclopropanes.

Recently, a unique oxidation/isomerisation of vinylidenecyclopropanes to dimethylenecyclopropanes using the system TPAP(cat.)/NMO has been discovered [209] (Scheme 54). The process proceeds with moderate to good yields. Other oxidants tested for the transformation gave complex mixtures or lower yields. Optimization of the process showed that chloroform or chloroform/CH_2_Cl_2_ mixtures were the best solvents at low temperature. The process works well for various *gem*-aryl-disubstituted cyclopropanes. The proposed mechanism is shown in Scheme 55. The sequence begins with the usual ester formation. Hydrogen transfer to the oxo-ruthenium portion then induces the cyclopropane ring opening to give intermediate **75** that recyclizes to give the final product. The proposed mechanism also explains the formation of minor amounts of the *E* isomer.

## 4. Conclusions

In conclusion, ruthenium tetroxide chemistry has further been developed in the last years. The most part of the recent work has been carried out on unsaturated substrates, and in particular on alkenes. Some processes have been satisfactorily improved. For example, RuO_4_ is more and more emerging as a useful alternative to osmium tetroxide in the dihydroxylation of alkenes filling the lack of, or the insufficient, reactivity shown by the latter on some substrates. Most of the protecting groups used in organic synthesis are tolerant to the employed reaction conditions and stereogenic centres, even adjacent to the reacting functional group, are generally unaffected. The reactions are fast and the differential reactivity of various functional groups [71] allows for the selective oxidation of only one of the diverse functionalities present, even avoiding, in some cases, protection/deprotection steps. A major limitation of the ruthenium tetroxide chemistry is currently represented by the impossibility of using asymmetric versions of the reactions catalyzed by this oxidant due to the strong oxidizing power of RuO_4_ that appears to be incompatible with the use of most of the known chiral ligands. Nonetheless, auxiliary-based diastereoselective alkene dihydroxylation procedures have been developed. On the other hand, perruthenate chemistry has also expanded further. New TPAP reactions have been discovered that enlarge the previously known TPAP reactivity. TPAP-catalyzed aerobic oxidations are a still developing research area and is presumable that further advances towards green processes will be realized in the future. An overview of some of important oxidative transformations mediated by other metal-oxo species, strictly related to RuO_4_ oxidation processes, has also been presented.
